# GC–MS analysis of phytoconstituents from *Amomum nilgiricum* and molecular docking interactions of bioactive serverogenin acetate with target proteins

**DOI:** 10.1038/s41598-020-73442-0

**Published:** 2020-10-02

**Authors:** Narasimhamurthy Konappa, Arakere C. Udayashankar, Soumya Krishnamurthy, Chamanalli Kyathegowda Pradeep, Srinivas Chowdappa, Sudisha Jogaiah

**Affiliations:** 1 Department of Studies in Biotechnology, University of Mysore, 570 006, Manasagangotri, Mysuru, Karnataka India; 2 Department of Microbiology, Field Marshal K. M. Cariappa College, A Constituent College of Mangalore University, Madikeri, 571201 Karnataka India; 3 Department of Microbiology and Biotechnology, Jnanabharathi Campus, Bangalore University, Bengaluru, 560 056 Karnataka India; 4 Laboratory of Plant Healthcare and Diagnostics, PG Department of Biotechnology and Microbiology, Karnataka University, Dharwad, Karnataka India

**Keywords:** Biochemistry, Biotechnology, Drug discovery, Microbiology, Plant sciences

## Abstract

*Amomum nilgiricum* is one of the plant species reported from Western Ghats of India, belonging to the family *Zingiberaceae*, with ethno-botanical values, and is well-known for their ethno medicinal applications. In the present investigation, ethyl acetate and methanol extracts of *A. nilgiricum* were analyzed by Fourier transform infrared spectrometer (FTIR) and gas chromatography-mass spectrometry (GC–MS) to identify the important functional groups and phytochemical constituents. The FTIR spectra revealed the occurrence of functional characteristic peaks of aromatic amines, carboxylic acids, ketones, phenols and alkyl halides group from leaf and rhizome extracts. The GC–MS analysis of ethyl acetate and methanol extracts from leaves, and methanol extract from rhizomes of *A. nilgiricum* detected the presence of 25 phytochemical compounds. Further, the leaf and rhizome extracts of *A. nilgiricum* showed remarkable antibacterial and antifungal activities at 100 mg/mL. The results of DPPH and ferric reducing antioxidant power assay recorded maximum antioxidant activity in *A. nilgiricum* methanolic leaf extract. While, ethyl acetate leaf extract exhibited maximum α-amylase inhibition activity, followed by methanolic leaf extract exhibiting aldose reductase inhibition. Subsequently, these 25 identified compounds were analyzed for their bioactivity through in silico molecular docking studies. Results revealed that among the phytochemical compounds identified, serverogenin acetate might have maximum antibacterial, antifungal, antiviral, antioxidant and antidiabetic properties followed by 2,4-dimethyl-1,3-dioxane and (1,3-^13^C_2_)propanedioic acid. To our best knowledge, this is the first description on the phytochemical constituents of the leaves and rhizomes of *A. nilgiricum*, which show pharmacological significance, as there has been no literature available yet on GC–MS and phytochemical studies of this plant species. The in silico molecular docking of serverogenin acetate was also performed to confirm its broad spectrum activities based on the binding interactions with the antibacterial, antifungal, antiviral, antioxidant and antidiabetic target proteins. The results of the present study will create a way for the invention of herbal medicines for several ailments by using *A. nilgiricum* plants, which may lead to the development of novel drugs.

## Introduction

Modern-day synthetic and chemical drugs are often explored with hesitate as they exhibit side effects^1^, while traditional herbals are gaining huge interests as they are more natural, environment-friendly and devoid of side effects^2^. Hence, with all the benefits of modern synthetic medicines, people have still preferred plant-based natural medicines over synthetic medicines^3^. Most of the medicinal plants are distinctive in their ability to treat, as well as to cure various human ailments owing to the contribution of various valuable phytoconstituents present in different plant parts^3–5^. In India, from ancient time, different parts of medicinal plants (~ 80,000 species) have been used as traditional medicines in different systems of Indian medicines for treatments of various diseases^6^. At present, about 25% of the active constituents have been identified from medicinal plants, which have been used as prescribed medicines^7^. Certain reports have estimated that over 25,000 of actual plant-based formulations are available in the Indian systems of folk and traditional medicine, which are prescribed by about 1.5 million practitioners in preventive, persuasive and healing applications^7,8^.

Various bioactive compounds of medicinal plants exhibit stimulating pharmacological actions like antibacterial, antifungal, anticancer, anti-inflammatory and antioxidant properties^3,4,9^. The potential of these bioactive compounds should be analyzed for their candidature in treatments of various ailments^4,7^. Plant-based medicines are often prepared from crude plant extracts comprising of complex mixture of different phytochemicals^7^. These phytochemicals have unique and complex structures, and are used in treating prolonged as well as contagious diseases^2,7^. An enormous pool of bioactive secondary metabolites exists in various plant species, but merely a small proportion of them have been examined and sustained to be significant source of bioactive agents. In the search for new compounds, and also for quality control, development of suitable screening methods is very important^10^. Extractions and characterizations of numerous such bioactive compounds from various medicinal plants have led to the delivery of certain medicines with high-activity profile^11^.

The initial screening of medicinal plants by spectrometric and chromatographic methods provides basic information on chemical and pharmacological activities, which helps to select the biologically active plants^12^. In recent years, Fourier-transform infrared (FTIR) and gas chromatography-mass spectrometry (GC–MS) has commonly been employed for detection of functional groups and identification of various bioactive therapeutic compounds that are present in medicinal plants^13,14^. GC–MS is one of the best, fast and accurate techniques to detect various compounds, including alcohols, alkaloids, nitro compounds, long chain hydrocarbons, organic acids, steroids, esters and amino acids^15^, and requires a small volume of plant extracts. Hence, in the present study, GC–MS technique was adopted for detection and identification of phytochemical compounds present in the medicinal plant, *Amomum nilgiricum* belonging to Zingiberaceae family. *A. nilgiricum* is a recent plant species found in the Western Ghats of Kerala, India^16^. Zingiberaceae is generally known as a family of spicy plants, and numerous members of this family have been used in Ayurvedic and other natural systems of medicine in India^17^. This family includes 53 genera and 1200 species^18^ that are known as ornamentals in different parts of the world, and have been broadly used as medicinal, traditional, food and ornamental plants in many regions of Asia, of which about 250 species are represented in India^16,18^.

Many drugs have failed to enter market owing to their poor pharmacokinetic properties, which incurs huge losses to pharmaceutical companies^19^. Computer-aided tools have emerged as advanced methods for drug discovery, which can be applied to screen the drugs from phytochemicals found in various medicinal plants^20^. Computational prediction models, also known as predictive tools, which play an important part in the selection of methodologies directing pharmaceutical and technological research, and have also been used in in silico prediction of pharmacological, pharmacokinetic and toxicological performance^21^. Currently, molecular docking is an effective and inexpensive method for designing and testing the drugs. This technique provides information about drug receptor interactions that are useful to predict the binding orientation of drug candidates to their target proteins^22^. Besides, this approach helps in systemic study by introducing a molecule on the binding spot of the object macromolecule in a non-covalent fashion, leading to an accurate binding at the active sites of each ligand^23^.

Therefore, the present study focuses on identification of bioactive compounds from ethyl acetate and methanol extracts from *A. nilgiricum* leaves, and methanol extract from *A. nilgiricum* rhizomes by GC–MS analysis. We have also evaluated the efficacy of leaves and rhizome extracts of *A. nilgiricum* for their antimicrobial, antioxidant and antidiabetic assay. Subsequently, in silico molecular docking and computational molecular simulation was explored for analysis of the potential bioactive compounds for their antibacterial, antifungal, antiviral, antioxidant and antidiabetic (e.g. α-glucosidase, α-amylase and aldose reductase) activities. Findings suggest that out of 25 identified phytochemical compounds, serverogenin acetate ([3,12-diacetyloxy-10,14-dimethyl-13-oxo-15-(5-oxo-2H-furan-3-yl)-2-oxapentacyclo[9.7.0.01,3.05,10.014,18]octadecan-7-yl] acetate) might have the best pharmacological properties. This report helps predict the structure and formula of phytochemical components present in various solvent extracts of *A. nilgiricum* plants.

## Results

### Preliminary phytochemical screening

The phytochemical study of extracts from *Amomum nilgiricum* leaves and rhizomes revealed a broad variety of phytochemicals. The key phytochemical components, such as flavonoids, anthocyanins, tannins, carbohydrates, alkaloids, cardiac glycosides, steroids, phenols, anthroquinone, leucoanthocyanin, diterpenes and saponins, were present in both the ethyl acetate and methanol extracts (Table 1).

**Table 1:** Phytochemical prescreening of *Amomum nilgiricum* rhizome and leaf extracts. The marks ‘ + ’ and ‘ − ’ indicate ‘present’ and ‘absent’, respectively.

Phytochemicals	Leaf extract		Rhizome extract
	Methanol	Ethyl acetate	Methanol
Carbohydrates	+	+	+
Flavonoids	+	+	+
Cardiac glycosides	+	+	+
Anthocyanin	−	−	+
Tannins	+	+	+
Alkaloids	+	+	+
Saponins	−	−	+
Steroids	+	+	+
Terpenoids	−	+	−
Phenols	+	+	+
Leucoanthocyanin	+	+	+
Proteins and amino acids	+	+	−
Diterpenes	+	+	+
Anthroquinone	−	−	+

### Fourier transform infrared spectrometer (FTIR)

**Figure 1:**
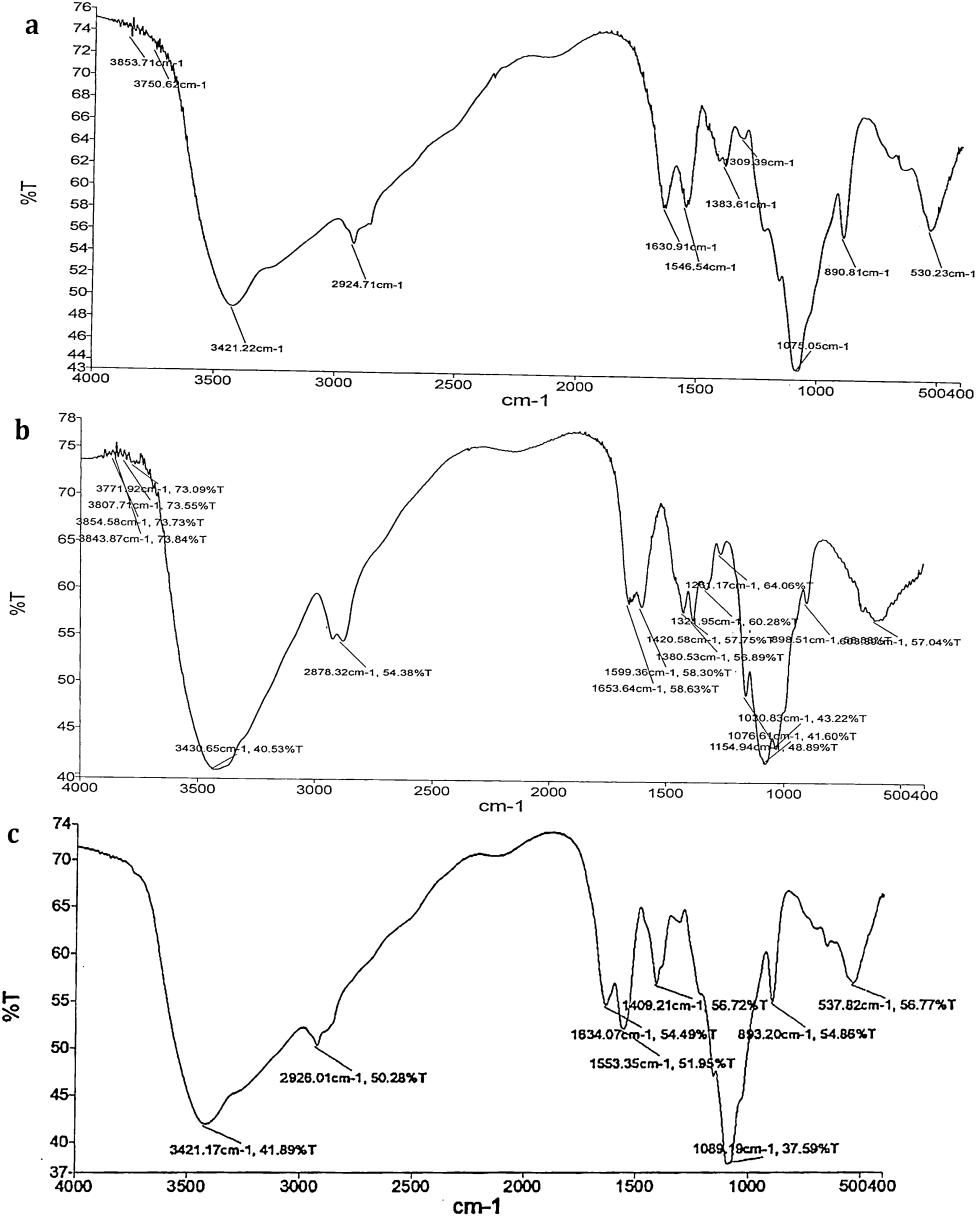
(**a**) FTIR spectrum from methanolic leaf extract of *Amomum nilgiricum*. (**b**) FTIR spectrum from ethyl acetate leaf extract of *Amomum nilgiricum*. (**c**) FTIR spectrum from methanolic rhizome extract of *Amomum nilgiricum.*

The results of FTIR spectra confirmed the presence of functional groups in methanolic leaf extract of *A. nilgiricum* with peaks at 3421.22 cm^−1^ (alcohols, phenols), 2924.71 cm^−1^ (alkanes), 1630.91 cm^−1^ (amines), 1546.54 cm^−1^ (alkanes), 1309.39 cm^−1^ (alcohols, carboxylic acids, esters, ethers), 1383.61 cm^−1^ (alkanes), 1075.05 cm^−1^ (aliphatic amines), 890.81 cm^−1^ (aromatics) and 530.23 cm^−1^ (alkyl halides) (Fig. 1a). The peaks at 3854.58 cm^−1^, 3843.87 cm^−1^, 3807.71 cm^−1^ (non-bonded, alcohols, phenols), 3771.92 cm^−1^ (carboxylic acids), 3430.65 cm^−1^ (alcohols, phenols), 2878.32 cm^−1^ (alkanes), 1653.64 cm^−1^ (alkenes), 1599.36 cm^−1^ (aromatics), 1420.58 cm^−1^ (aromatics), 1380.53 cm^−1^ (alkanes), 1321.95 cm^−1^ (alcohols, carboxylic acids, esters, ethers), 1261.17 cm^−1^ (aromatic amines), 1154.94 cm^−1^ (alkyl halides), 1076.61 cm^−1^ (aliphatic amines), 1030.83 cm^−1^ (aliphatic amines) and 608.52 cm^−1^ (alkyl halides) determined the existence of functional groups ethyl acetate leaf extract (Fig. 1b).

Likewise, the FTIR spectrum from *A. nilgiricum* methanolic rhizome extract showed the presence of functional groups with peaks at 3421.17 cm^−1^ (alcohols, phenols), 2926.01 cm^−1^ (alkanes), 1634.07 cm^−1^ (amines), 1553.35 cm^−1^ (nitro compounds), 1409.21 cm^−1^ (aromatics), 1089.19 cm^−1^ (aliphatic amines), 893.20 cm^−1^ (aromatics), 537.82 cm^−1^ (alkyl halides) (Fig. 1c).

### Gas chromatography-mass spectrometry (GC–MS) analysis

The GC–MS chromatogram of methanol and ethyl acetate leaf extracts of *A. nilgiricum* recorded a total of 15 peaks corresponding to the bioactive compounds that were recognized by relating their peak retention time, peak area (%), height (%) and mass spectral fragmentation patterns to that of the known compounds described by the National Institute of Standards and Technology (NIST) library. Results revealed that 9 and 6 compounds were identified in methanol and ethyl acetate *A. nilgiricum* leaf extracts, respectively (Table 2 & 3). The GC–MS chromatogram of the methanol extract from *A. nilgiricum* rhizomes recorded 10 peaks (Table 4). Overall, the structure of 25 phytocompounds identified in the ethyl acetate and methanol extracts from leaves, and from the methanol extract from rhizomes of *A. nilgiricum* are presented in Table 5 along with their retention time.

**Table 2:** Phytochemical constituents identified in the methanol leaf extract of *Amomum nilgiricum* using gas chromatography-mass spectrometry.

Sl. No	CAS	Name of the compound	Molecular formula	Molecular weight	Peak area (%)
1	5699–55-8	5-aminooxypentanoic acid	C_5_H_11_O_3_N	133.14	4.082
2	40,838–15-1	3,7,9-trioxatricyclo[4.2.1.0^2,4^]nonane	C_6_H_8_O_3_	128.13	19.379
3	78,721–87-6	1-(2,4,4-trimethylpentan-2-yl)-4-[4-(2,4,4-trimethylpentan-2-yl)phenoxy]benzene	C_17_H_30_OSi	278.50	3.129
4	17,151–09-6	Trimethyl-(2-trimethylsilylphenyl)silane	C_12_H_22_Si_2_	222.47	3.171
5	900,161–21-8	3,5-bis(trimethylsilyl)cyclohepta-2,4,6-trien-1-one	C_13_H_22_OSi_2_	250.48	4.369
6	900,283–56-8	Trimethyl-[4-[2-methyl-4-(4-trimethylsilyloxyphenyl)pent-4-en-2-yl]phenoxy]silane	C_24_H_36_O_2_Si_2_	412.7	38.631
7	70,244–15-4	Trimethyl-[4-[4-(4-trimethylsilyloxyphenyl)hexan-3-yl]phenoxy]silane	C_24_H_38_O_2_Si_2_	414.72	16.246
8	13,183–70-5	Trimethyl-[[4-(trimethylsilylmethyl)phenyl]methyl]silane	C_14_H_26_Si_2_	250.47	6.405
9	1873–88-7	1,1,1,3,5,5,5-heptamethyltrisiloxane	C_7_H_22_O_2_Si_3_	221.5	4.588

*CAS* chemical abstract service, *Sl. No.* serial number.

**Table 3:** Phytochemical constituents identified in the ethyl acetate leaf extract of *Amomum nilgiricum* using gas chromatography-mass spectrometry.

Sl. no	CAS	Name of the compound	Molecular formula	Molecular weight	Peak area (%)
1	629–89-0	Octadec-1-yne	C_18_H_34_	250.5	2.852
2	63,922–50-9	3,4-heptadien-2-one, 3-cyclopentyl-6-methyl-	C_13_H_20_O	192.3	3.027
3	900,253–62-2	[3,12-diacetyloxy-10,14-Dimethyl-13-oxo-15-(5-oxo-2H-furan-3-yl)-2-oxapentacyclo[9.7.0.01,3.05,10.014,18]octadecan-7-yl] acetate	C_29_H_36_O_10_	544.6	82.105
4	78,721–87-6	Trimethyl-[4-(2,4,4-trimethylpentan-2-yl)phenoxy]silane	C_17_H_30_OSi	278.51	3.757
5	17,151–09-6	Trimethyl-(2-trimethylsilylphenyl)silane	C_12_H_22_Si_2_	222.47	4.706
6	900,161–21-8	3,5-bis(trimethylsilyl)cyclohepta-2,4,6-trien-1-one	C_13_H_22_OSi_2_	250.48	3.553

*CAS* chemical abstract service, *Sl. No.* serial number.

**Table 4:** Phytochemical constituents identified in the methanol rhizome extract of *Amomum nilgiricum* using gas chromatography-mass spectrometry.

Sl. no	CAS	Name of the compound	Molecular formula	Molecular weight	Peak area (%)
1	617–35-6	Ethyl 2-oxopropanoate	C_5_H_8_O_3_	116.11	2.271
2	141–82-2	(1,3-^13^C_2_)propanedioic acid	C_3_H_4_O_4_	104.06	6.142
3	900,143–55-6	2-butyl-4-[(E)-pentadec-4-enyl]-1,3,2-dioxaborinan-5-amine	C_22_H_44_O_2_NB	365.4	18.518
4	766–20-1	2,4-dimethyl-1,3-dioxane	C_6_H_12_O_2_	116.16	12.222
5	900,270–67-7	5-amino-6-nitroso-1*H*-pyrimidine-2,4-dione	C_4_H_4_O_3_N_4_	156.1	9.325
6	900,129–98-9	(2*R*,4*S*,5*S*,6*R*)-3,8,9-trioxatricyclo[4.2.1.0^2,4^]nonan-5-ol	C_6_H_8_O_4_	144.12	33.323
7	900,129–96-9	(1*R*,2*S*,4*R*,5*R*)-3,7,9-trioxatricyclo[4.2.1.0^2,4^]nonan-5-ol	C_6_H_8_O_4_	144.12	3.300
8	900,129–98-0	(2*S*,4*S*,5*R*,6*R*)-3,8,9-trioxatricyclo[4.2.1.0^2,4^]nonan-5-ol	C_6_H_8_O_4_	144.12	9.621
9	23,293–50-7	5-methylhex-3-yn-2-ol	C_7_H_12_O	112.17	2.818
10	74,367–37-6	Trimethylsilyl tetracosanoate	C_27_H_56_O_2_Si	440.8	2.460

*CAS* chemical abstract service, *Sl. No*. serial number.

**Table 5:** Structures and retention time (min) of phytochemical constituents identified in the ethyl acetate and methanol extracts of *Amomum nilgiricum* leaves, and the methanol extract of *A. nilgiricum* rhizomes using gas chromatography-mass spectrometry.

Sl. no	Retention time	Name of the compound	Structure
1	RT-20.986	5-aminooxypentanoic acid	C(CCON)CC(=O)O
2	RT-21.916	3,7,9-trioxatricyclo[4.2.1.0^2,4^]nonane	C_1_C_2_C(O_2_)C_3_COC_1_O_3_
3	RT-27.368	1-(2,4,4-trimethylpentan-2-yl)-4-[4-(2,4,4-trimethylpentan-2-yl)phenoxy]benzene	CC(C)(C)CC(C)(C)C_1_CCC(CC_1_)O[Si](C)(C)C
4	RT-27.753	Trimethyl-(2-trimethylsilylphenyl)silane	C[Si](C)(C)C_1_CCCCC_1_[Si](C)(C)C
5	RT-27.898	3,5-bis(trimethylsilyl)cyclohepta-2,4,6-trien-1-one	[SiH_3_]C_1_CC(=O)CCC([SiH_3_])C_12_
6	RT-28.043	Trimethyl-[4-[2-methyl-4-(4-trimethylsilyloxyphenyl)pent-4-en-2-yl]phenoxy]silane	CC(C)(CC(=C)C1=CC=C(C=C_1_)O[Si](C)(C)C)C_2_=CC=C(C=C_2_)O[Si](C)(C)C
7	RT-28.288	Trimethyl-[4-[4-(4-trimethylsilyloxyphenyl)hexan-3-yl]phenoxy]silane	CCC(C_1_CCC(CC_1_)O[Si](C)(C)C)C(CC)C_2_CCC(CC_2_)O[Si](C)(C)C_1_
8	RT-28.489	Trimethyl-[[4-(trimethylsilylmethyl)phenyl]methyl]silane	[SiH_3_]C_1_CCC([SiH_3_])CC
9	RT-28.589	1,1,1,3,5,5,5-heptamethyltrisiloxane	C[SIH](O[Si](C)(C)C)O[Si](C)(C)C
10	RT-19.950	Octadec-1-yne	CCCCCCCCCCCCCCCCC#C
11	RT-21.086	3,4-heptadien-2-one, 3-cyclopentyl-6-methyl-	CC(C)C=C=C(C1CCCC1)C(=O)C
12	RT-21.891	[3,12-diacetyloxy-10,14-dimethyl-13-oxo-15-(5-oxo-2H-furan-3-yl)-2-oxapentacyclo[9.7.0.01,3.05,10.014,18]octadecan-7-yl] acetate	CC(=O)OC_1_CCC_2_(C(C_1_)CC_3_(C_4_(C_2_C(C(=O)C_5_(C_4_CCC_5_C_6_=CC(=O)OC_6_)C)OC(=O)C)O_3_)OC(=O)C)C
13	RT-27.098	Trimethyl-[4-(2,4,4-trimethylpentan-2-yl)phenoxy]silane	CC(C)(C)CC(C)(C)C_1_CCC(CC_1_)O[Si](C)(C)C
14	RT-27.718	Trimethyl-(2-trimethylsilylphenyl)silane	C[Si](C)(C)C_1_CCCCC_1_[Si](C)(C)C
15	RT-27.783	3,5-bis(trimethylsilyl)cyclohepta-2,4,6-trien-1-one	C[Si](C)(C)C_1_=CC(=CC(=O)C=C_1_)[Si](C)
16	RT-4.759	Ethyl 2-oxopropanoate	CCOC(=O)C(=O)C
17	RT-7.880	(1,3-^13^C_2_)propanedioic acid	C(C(=O)O)C(=O)O
18	RT-15.473	2-butyl-4-[(E)-pentadec-4-enyl]-1,3,2-dioxaborinan-5-amine	B_1_(OCC(C(O_1_)CCC/C=C/CCCCCCCCCC)N)CCCC
19	RT-16.399	2,4-dimethyl-1,3-dioxane	CC_1_CCOC(O_1_)C
20	RT-16.989	5-amino-6-nitroso-1*H*-pyrimidine-2,4-dione	C1(C([NH]C(=O)[NH]C_1_=O)N=O)N
21	RT-18.084	(2*R*,4*S*,5*S*,6*R*)-3,8,9-trioxatricyclo[4.2.1.0^2,4^]nonan-5-ol	C_1_C_2_C(C_3_C(O_3_)C(O_1_)O_2_)O
22	RT-18.635	(1*R*,2*S*,4*R*,5*R*)-3,7,9-trioxatricyclo[4.2.1.0^2,4^]nonan-5-ol	C_1_C_2_C_3_C(O_3_)C(C(O_1_)O_2_)O
23	RT-18.755	(2*S*,4*S*,5*R*,6*R*)-3,8,9-trioxatricyclo[4.2.1.0^2,4^]nonan-5-ol	C_1_C_2_C(C3C(O_3_)C(O_1_)O_2_)O
24	RT-19.430	5-methylhex-3-yn-2-ol	CC(C)C#CC(C)O
25	RT-21.911	Trimethylsilyl tetracosanoate	CCCCCCCCCCCCCCCCCCCCCCCC(=O)O[Si](C)(C)C

*Sl. No*. serial number.

**Figure 2:**
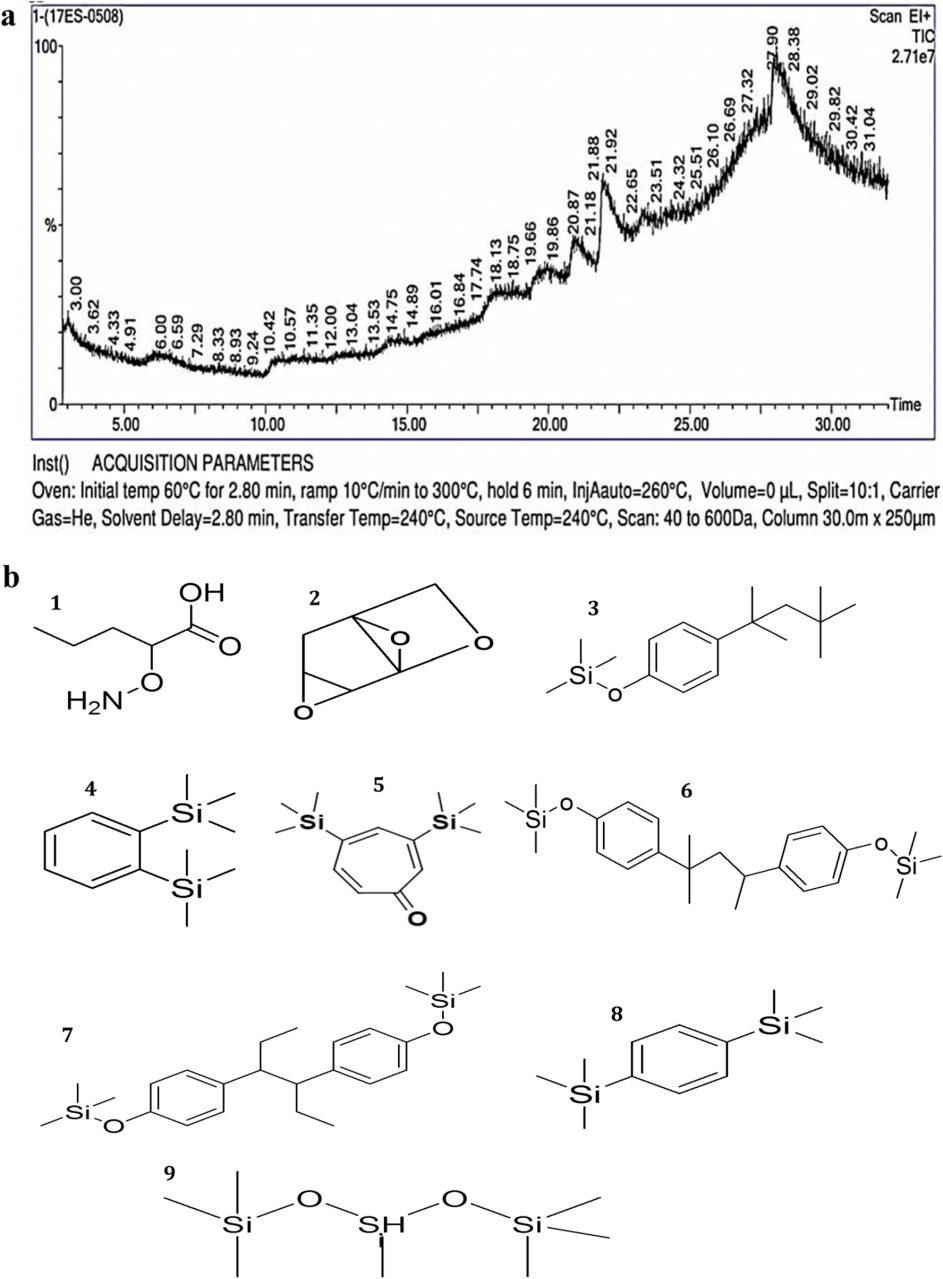
(**a**) Phytoconstituents detected in the methanol leaf extract of *Amomum nilgiricum* using gas chromatography-mass spectrometry*.* (**b**). chemical structure of nine phytocompounds identified based on the retention time and peak area, namely (1) 5-aminooxypentanoic acid (20.986 retention time and 4.082% peak area), (2) 3,7,9-trioxatricyclo[4.2.1.0^2,4^]nonane (21.916 retention time and 19.379% peak area), (3) 1-(2,4,4-trimethylpentan-2-yl)-4-[4-(2,4,4-trimethylpentan-2-yl)phenoxy]benzene (27.368 retention time and 3.129% peak area), (4) trimethyl-(2-trimethylsilylphenyl)silane (27.753 retention time and 3.171% peak area), (5) 3,5-bis(trimethylsilyl)cyclohepta-2,4,6-trien-1-one, (27.898 retention time and 4.369% peak area), (6) trimethyl-[4-[2-methyl-4-(4-trimethylsilyloxyphenyl)pent-4-en-2-yl]phenoxy]silane (28.043 retention time and 38.631% peak area), (7) trimethyl-[4-[4-(4-trimethylsilyloxyphenyl)hexan-3- (28.288 retention time and 16.246% peak area), (8) trimethyl-[[4-(trimethylsilylmethyl)phenyl]methyl]silane (28.489 retention time and 6.405% peak area) and (9) 1,1,1,3,5,5,5-heptamethyltrisiloxane (28.589 retention time and 4.588% peak area).

**Figure 3:**
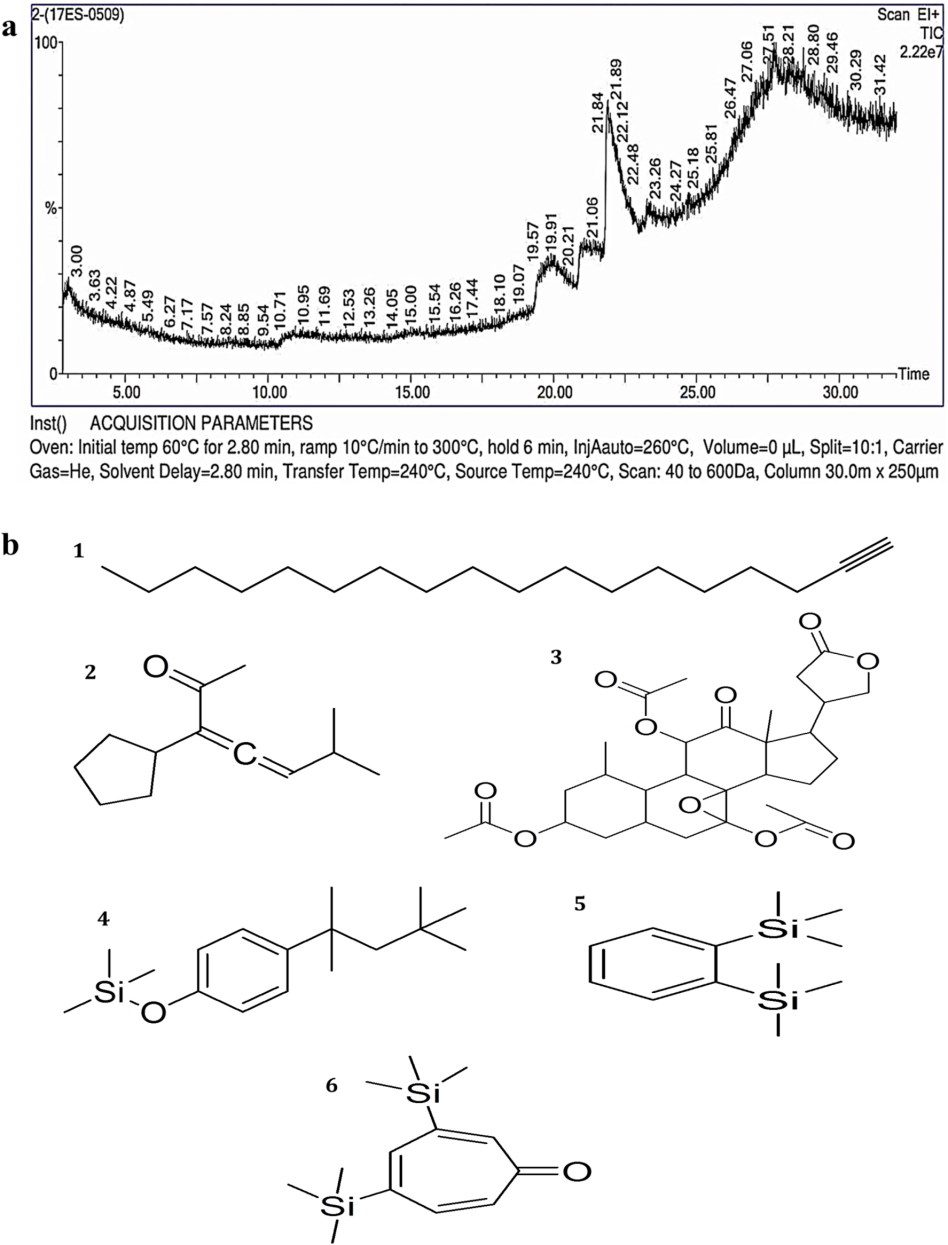
(**a**)**.** Phytoconstituents detected in the ethyl acetate extract from *Amomum nilgiricum* leaves using gas chromatography-mass spectrometry. (**b**). Chemical structure of six phytocompounds identified based on the retention time and peak area, namely (1) octadec-1-yne, (19.950 retention time and 2.852% peak area), (2) 3,4-heptadien-2-one, 3-cyclopentyl-6-methyl- (21.086 retention time and 3.027% peak area), (3) [3,12-diacetyloxy-10,14-dimethyl-13-oxo-15-(5-oxo-2H-furan-3-yl)-2-oxapentacyclo[9.7.0.01,3.05,10.014,18]octadecan-7-yl] acetate (21.891 retention time and 82.105% peak area), (4) trimethyl-[4-(2,4,4-trimethylpentan-2-yl)phenoxy]silane (27.098 retention time and 3.757% peak area), (5) trimethyl-(2-trimethylsilylphenyl)silane (27.718 retention time and 4.706% peak area) and (6) 3,5-bis(trimethylsilyl)cyclohepta-2,4,6-trien-1-one (27.783 retention time and 3.553% peak area).

The phytoconstituents in the methanol leaf extract of *A. nilgiricum* were found to be 5-aminooxypentanoic acid, 3,7,9-trioxatricyclo[4.2.1.0^2,4^]nonane, 1-(2,4,4-trimethylpentan-2-yl)-4-[4-(2,4,4-trimethylpentan-2-yl)phenoxy]benzene, trimethyl-(2-trimethylsilylphenyl)silane, 3,5-bis(trimethylsilyl)cyclohepta-2,4,6-trien-1-one, trimethyl-[4-[2-methyl-4-(4-trimethylsilyloxyphenyl)pent-4-en-2-yl]phenoxy]silane, trimethyl-[4-[4-(4-trimethylsilyloxyphenyl)hexan-3-yl]phenoxy]silane, trimethyl-[[4-(trimethylsilylmethyl)phenyl]methyl]silane and 1,1,1,3,5,5,5-heptamethyltrisiloxane (Fig. 2a,b, Table 2). The phytoconstituents in ethyl acetate leaf extract of *A. nilgiricum* were octadec-1-yne, 3,4-heptadien-2-one, 3-cyclopentyl-6-methyl-, [3,12-diacetyloxy-10,14-dimethyl-13-oxo-15-(5-oxo-2H-furan-3-yl)-2-oxapentacyclo[9.7.0.01,3.05,10.014,18]octadecan-7-yl] acetate, trimethyl-[4-(2,4,4-trimethylpentan-2-yl)phenoxy]silane, trimethyl-(2-trimethylsilylphenyl)silane, and 3,5-bis(trimethylsilyl)cyclohepta-2,4,6-trien-1-one (Fig. 3a,b, Table 3).

**Figure 4:**
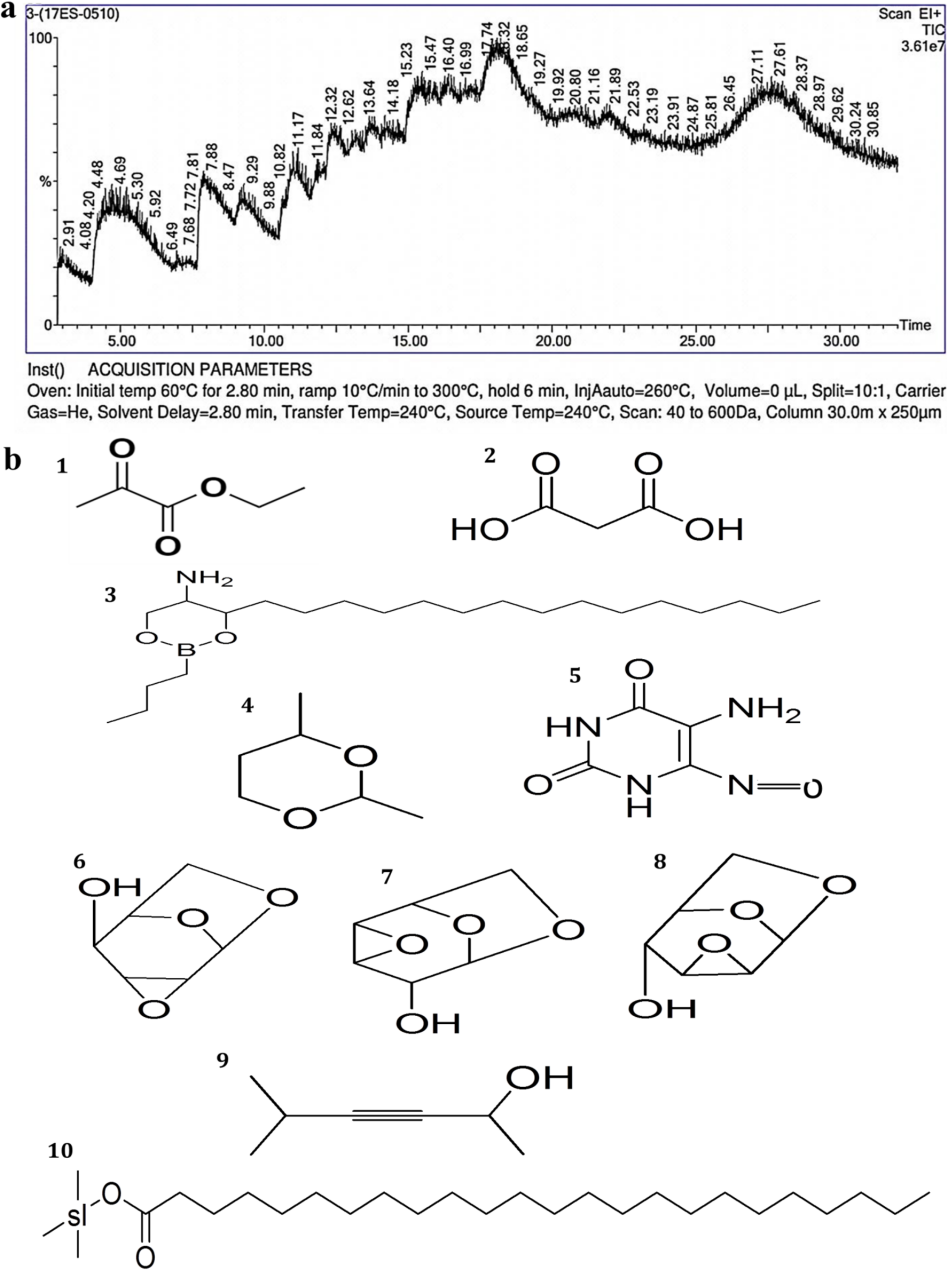
(**a**)**.** Phytoconstituents detected in the methanol rhizome extract of *Amomum nilgiricum* using gas chromatography-mass spectrometry. (**b**). Chemical structure of ten phytocompounds identified based on the retention time and peak area, namely (1) ethyl 2-oxopropanoate (4.759 retention time and 2.271% peak area), (2) (1,3-^13^C_2_)propanedioic acid (7.880 retention time and 6.142% peak area), (3) 2-butyl-4-[(E)-pentadec-4-enyl]-1,3,2-dioxaborinan-5-amine (15.473 retention time and 18.518% peak area), (4) 2,4-dimethyl-1,3-dioxane (16.399 retention time and 12.222% peak area), (5) 5-amino-6-nitroso-1*H*-pyrimidine-2,4-dione (16.989 retention time and 9.325% peak area), (6) (2*R*,4*S*,5*S*,6*R*)-3,8,9-trioxatricyclo[4.2.1.0^2,4^]nonan-5-ol (18.084 retention time and 33.323% peak area), (7) (1*R*,2*S*,4*R*,5*R*)-3,7,9-trioxatricyclo[4.2.1.0^2,4^]nonan-5-ol (18.635 retention time and 3.300% peak area), (8) (2*S*,4*S*,5*R*,6*R*)-3,8,9-trioxatricyclo[4.2.1.0^2,4^]nonan-5-ol (18.755 retention time and 9.621% peak area), (9) 5-methylhex-3-yn-2-ol (19.430 retention time and 2.818% peak area) and (10) trimethylsilyl tetracosanoate (21.911 retention time and 2.460% peak area).

The GC–MS chromatogram of methanol extract from *A. nilgiricum* rhizome recorded 10 peaks corresponding to bioactive compounds, which were recognized by comparing its mass spectra along with their analogs reported in the NIST library (Table 4). The major components present in *A. nilgiricum* were ethyl 2-oxopropanoate, (1,3-^13^C_2_)propanedioic acid, 2-butyl-4-[(E)-pentadec-4-enyl]-1,3,2-dioxaborinan-5-amine, 2,4-dimethyl-1,3-dioxane, 5-amino-6-nitroso-1*H*-pyrimidine-2,4-dione, (2*R*,4*S*,5*S*,6*R*)-3,8,9-trioxatricyclo[4.2.1.0^2,4^]nonan-5-ol, (2*S*,4*S*,5*R*,6*R*)-3,8,9-trioxatricyclo[4.2.1.0^2,4^]nonan-5-ol, 5-methylhex-3-yn-2-ol and trimethylsilyl tetracosanoate (Fig. 4a,b, Table 4).

### Antibacterial activity

**Figure 5:**
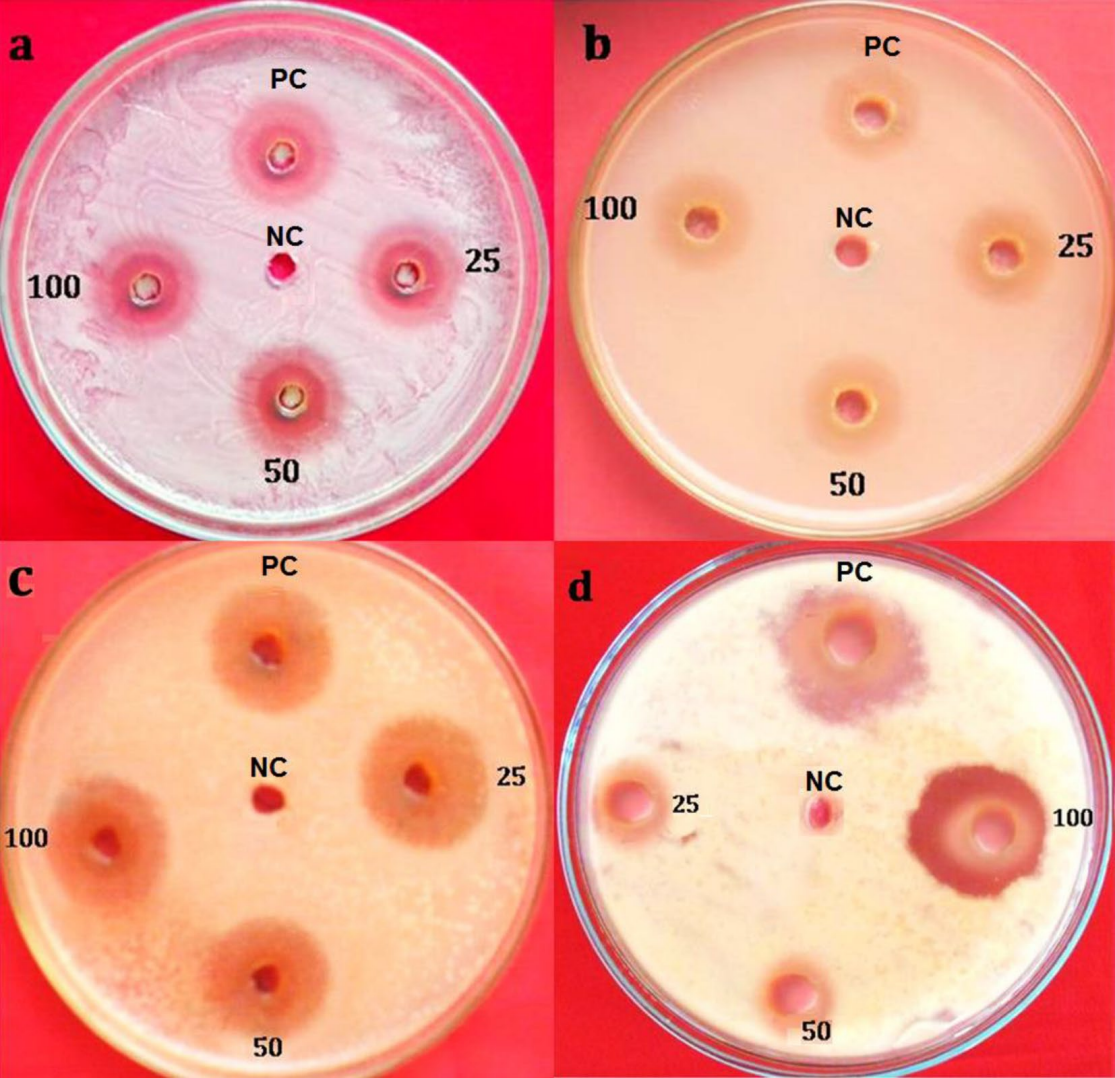
Antibacterial activity of leaf and rhizome extracts of *A. nilgiricum* at 25, 50 and 100 mg/ mL (**a**) Zone of inhibition from ethyl acetate leaf extract against *Pseudomonas aeruginosa* (**b**) Zone was from methanol leaf extract against *Pseudomonas aeruginosa.* (**c**) Zone of inhibition from ethyl acetate leaf extract against *R. solanacearum* (**d**) Zone of inhibition from methanolic rhizome extract against *Ralstonia solanacearum.* PC: Streptomycin (positive control); NC: solvent extract (negative control).

Antibacterial activities of leaf and rhizome extracts of *A. nilgiricum* were tested against different bacterial pathogens at different concentrations (25, 50 and 100 mg/mL). Maximum zone of inhibition of 21.43 mm was recorded with ethyl acetate leaf extract against *Pseudomonas aeruginosa* and highest zone of inhibition was observed for methanolic rhizome extract against *Ralstonia solanacearum* with 20.72 mm at 100 mg/ mL as compared to other tested concentrations (Fig. 5).

### Antifungal activity

**Figure 6:**
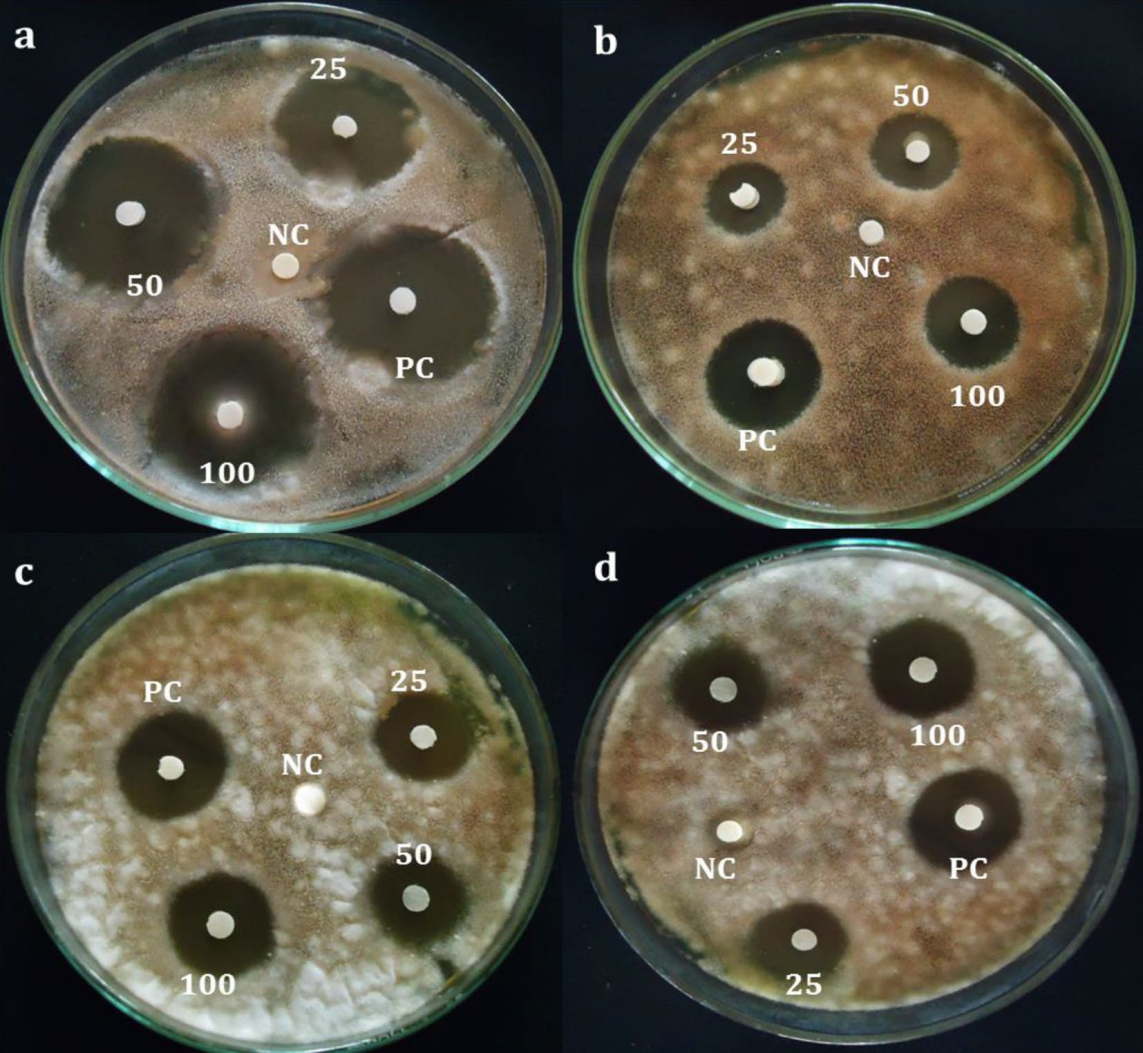
Antifungal activity of *Amomum nilgiricum* leaf and rhizome extracts at 25, 50 and 100 mg/mL (**a**) Zone of inhibition from ethyl acetate leaf extract against *Alternaria alternata* (**b**) methanol leaf extract against *Alternaria alternata.* (**c**) Ethyl acetate leaf extract against *Pyricularia oryzae*. (**d**) Methanolic rhizome extract against *Pyricularia oryzae.* PC: (Positive control) Nystatin; NC: Negative control (solvent).

The ethyl acetate leaf extract of *A. nilgiricum* recorded maximum zone of inhibition of 16.69 mm against *Alternaria alternate*. While, methanolic rhizome extract at 100 mg/mL showed inhibition of 16.32 mm against *Pyricularia oryzae* (16.32 mm) when compared to other tested concentrations used in the study (Fig. 6).

### Antioxidant activity

**Figure 7:**
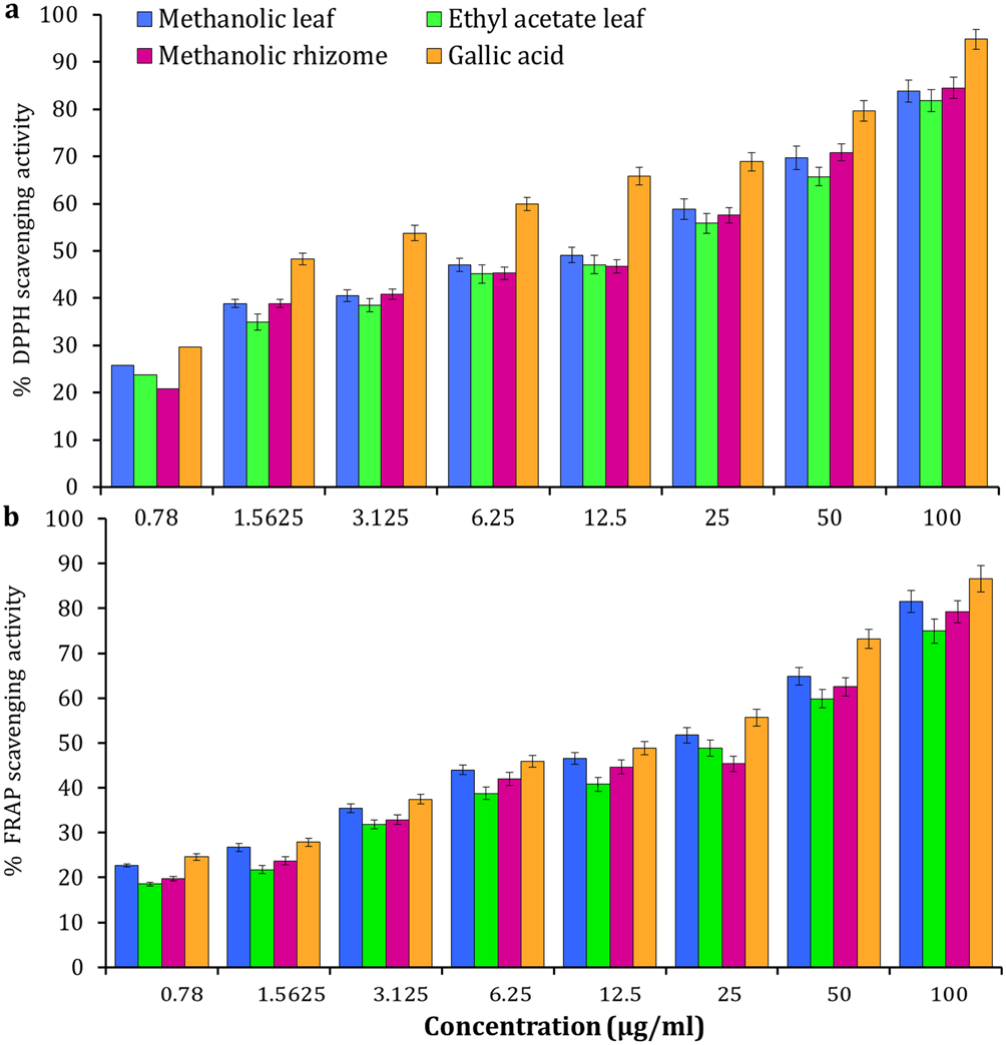
Antioxidant activity of *Amomum nilgiricum* leaf and rhizome extracts. (**a**) DPPH radical scavenging activity. (**b**) Ferric reducing antioxidant power scavenging activity.

In the present study, the different concentrations of leaf and rhizome extracts of *A. nilgiricum* were subjected to 2,2-diphenyl-1-picryl-hydrazyl-hydrate (DPPH) free radical scavenging method. The methanolic leaf extract was revealed maximum DPPH activity of 83.68%, while gallic acid, ethyl acetate leaf and methanolic rhizome extracts recorded about 94.85%, 81.86% and 84.53%, respectively (Fig. 7a). The methanolic leaf extract exhibited the highest DPPH radical scavenging activity with an IC_50_ of 50.65 µg/ml, followed by methanolic rhizome and ethyl acetate extracts with an IC_50_ values of 59.42 µg/ml and 78.54 µg/ml respectively, whereas the gallic acid exhibited IC_50_ values of 48 µg/ml. The percent Ferric reducing antioxidant power assay (FRAP) activity of leaf and rhizome extracts recorded 18.56 to 81.57% (Fig. 7b). Among extracts, methanolic leaf extract revealed a maximum FRAP activity with an IC_50_ value was 60.65 μg/ml, ethyl acetate leaf extract was showed IC_50_ value of 79.79 µg/ml and methanolic rhizome extracts was showed IC_50_ value of 73.42 µg/ml. The IC_50_ value of the extract was less than that of the standard.

### Antidiabetic activity

**Figure 8:**
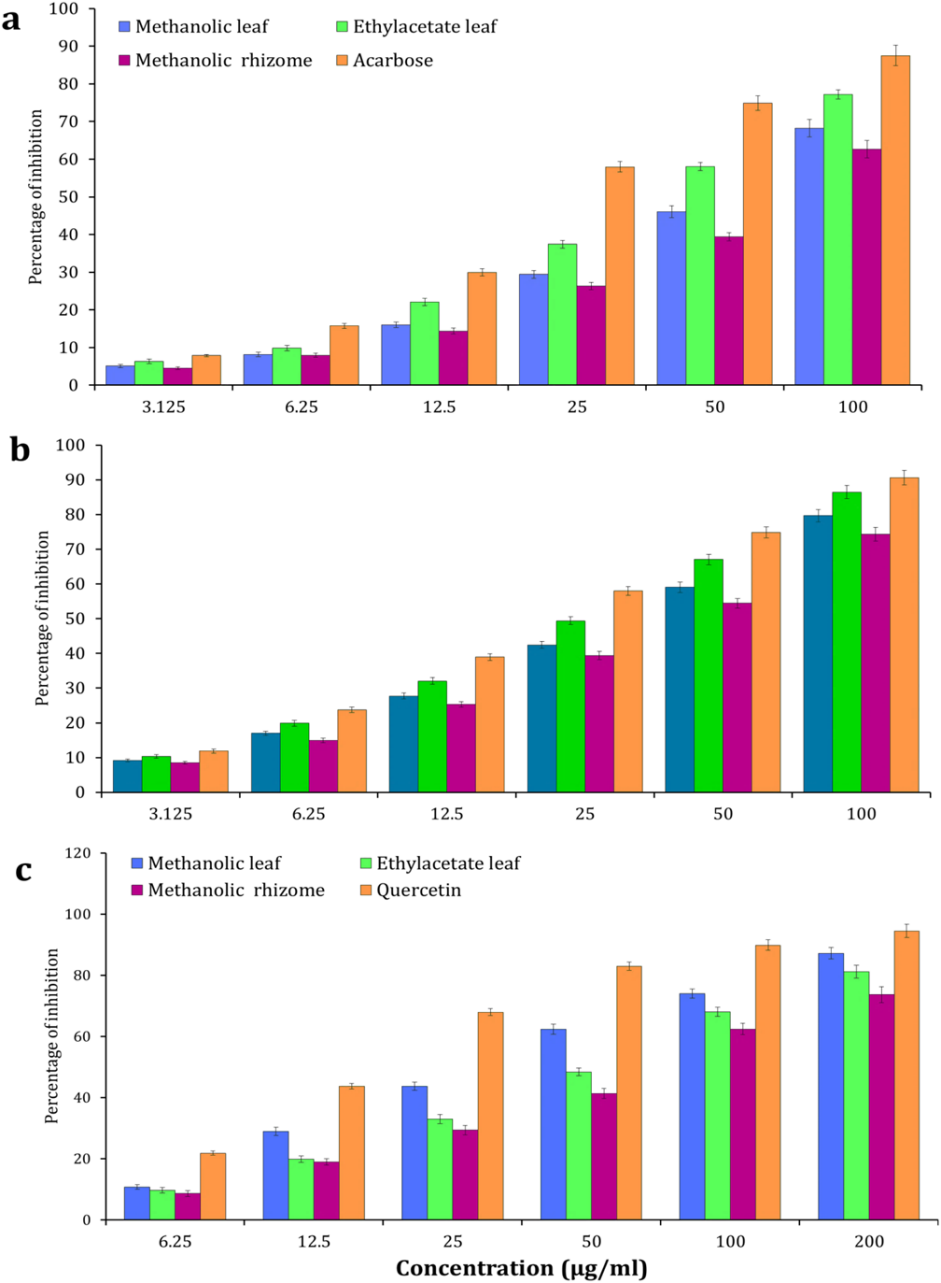
Antidiabetic enzymes activities of *Amomum nilgiricum* leaf and rhizome extracts. (**a**) α—amylase inhibition activity. (**b**) α—glucosidase inhibition activity. (**c**) aldose reductase inhibition activity.

A dose dependent inhibitory action on α-amylase activity was observed from the leaf and rhizome of *A. nilgiricum* extracts (Fig. 8a). Among the extracts, ethyl acetate extract exhibited maximum α- amylase inhibition with 77.22% (IC_50_ 76.53 µg/ml), followed by 68.22% (IC_50_ 89.43 µg/ml) recorded for methanolic leaf extract and methanolic rhizome extract showed 55.12% (IC_50_ 99.38 µg/ml). The standard Acarbose showed α-amylase inhibition of 87.17% (IC_50_ 0.32 μg/ml). A dose dependent activity of α-glucosidase enzyme inhibition was noticed with standard Acarbose (Fig. 8b). The ethyl acetate leaf extract exhibited maximum α-glucosidase inhibition was 85.26% (IC_50_ 72.54 µg/ml), followed by the methanol leaf extract with 78.34% (IC_50_ 87.32 µg/ml) and methanolic rhizome extract 71.66% (IC_50_ 99.46 µg/ml). While, standard Acarbose showed α-glucosidase inhibition of 89.54% (IC_50_ 0.46 μg). Among extracts, the methanolic leaf extract showed highest 87.22%inhibition of aldose reductaseas compared to other extracts (Fig. 8c). In case of ethyl acetate leaf extract and methanolic rhizome extracts, a inhibition of 81.31 and 73.66% of aldose reductase was documented in the present study. The aldose reductase inhibition with an IC_50_ value from ethyl acetate, methanol leaf and methanolic rhizome extracts revealed 77.54, 48.7 and 99.32 μg/ml respectively. While, Quercetin recorded best activity of 7.95 μg/ml with IC_50_ value.

### In silico studies

The GC–MS analysis revealed that *A. nilgiricum* leaf and rhizome extracts contained 25 bioactive compounds (Table 5). These phytocompounds were analyzed for activities against bacterial, fungal, viral, antioxidant and diabetic target proteins. The docking studies were carried out for phytoligands using the iGEMDOCK program to elucidate the binding affinities to the target proteins. The binding interaction and conformation of each phytocompound with each target protein were predicted and ranked based on the iGEMDOCK-computed lowest energy and total binding energy, respectively. The best binding conformation for each phytocompound with each target protein was determined, if it showed the lowest total binding energy among its different conformations (Table 6).

**Table 6:** Binding energies of 25 phytocompounds against bacterial, fungal, viral, antioxidant and diabetic (e.g. α-glucosidase, α-amylase and aldose reductase) target proteins.[3,12-diacetyloxy-10,14-dimethyl-13-oxo-15-(5-oxo-2H-furan-3-yl)-2-oxapentacyclo[9.7.0.01,3.05,10.014,18]octadecan-7-yl] acetate (red) showed the best binding conformations with tested target proteins.

Name of the compound	Target proteins						
	Antibacterial (kcal/mol)	Antifungal (kcal/mol)	Antiviral (kcal /mol)	Antioxidant (kcal/mol)	Aldo reductase (kcal/mol)	Alpha glucosidase (kcal/mol)	Alpha amylase (kcal/mol)
5-aminooxypentanoic acid	− 66.752	− 71.1418	− 69.0289	− 63.1516	− 68.1574	− 66.9723	− 71.7856
3,7,9-trioxatricyclo[4.2.1.0^2,4^]nonane	− 54.5983	− 57.2671	− 56.0637	− 53.3396	− 66.216	− 67.5377	− 57.9483
1-(2,4,4-trimethylpentan-2-yl)-4-[4-(2,4,4-trimethylpentan-2-yl)phenoxy]benzene	− 65.0987	− 68.3489	− 62.7397	− 57.2621	− 84.2382	− 77.612	− 66.8286
Trimethyl-(2-trimethylsilylphenyl)silane	− 56.2474	− 55.4126	− 55.5996	− 52.3413	− 68.2982	− 59.2383	− 55.877
3,5-bis(trimethylsilyl)cyclohepta-2,4,6-trien-1-one	− 72.7044	− 82.1708	− 68.961	− 75.8948	− 101.154	− 68.9778	− 72.3101
Trimethyl-[4-[2-methyl-4-(4-trimethylsilyloxyphenyl)pent-4-en-2-yl]phenoxy]silane	− 75.6489	− 73.6996	− 69.0353	− 66.5933	− 74.3881	− 75.7783	− 76.2131
Trimethyl-[4-[4-(4-trimethylsilyloxyphenyl)hexan-3-yl]phenoxy]silane	− 46.8452	− 52.8639	− 45.2415	− 42.2495	− 53.1494	− 53.0229	− 45.8263
Trimethyl-[[4-(trimethylsilylmethyl)phenyl]methyl]silane	− 46.4648	− 49.5256	− 53.544	− 51.1354	− 59.5502	− 56.6869	− 49.4279
1,1,1,3,5,5,5-heptamethyltrisiloxane	− 60.0582	− 57.4102	− 55.932	− 47.7934	− 57.7133	− 59.4908	− 55.2875
Octadec-1-yne	− 65.6492	− 81.3123	− 74.2114	− 62.2128	− 85.6565	− 69.7406	− 59.9751
3,4-heptadien-2-one, 3-cyclopentyl-6-methyl-	− 60.6587	− 67.5671	− 67.3529	− 59.9746	− 67.1473	− 63.9103	− 61.7223
Serverogenin acetate [3,12-diacetyloxy-10,14-dimethyl-13-oxo-15-(5-oxo-2H-furan-3-yl)-2-oxapentacyclo[9.7.0.01,3.05,10.014,18]octadecan-7-yl] acetate	− 103.145	− 105.789	− 90.5357	− 82.8571	− 137.034	− 94.6438	− 94.7775
Trimethyl-[4-(2,4,4-trimethylpentan-2-yl)phenoxy]silane	− 65.0283	− 75.0271	− 66.8266	− 61.3058	− 88.5434	− 77.6173	− 66.588
Trimethyl-(2-trimethylsilylphenyl)silane	− 55.7175	− 58.7707	− 56.8283	− 48.5849	− 59.4178	− 59.3893	− 55.4465
3,5-bis(trimethylsilyl)cyclohepta-2,4,6-trien-1-one	− 53.0653	− 59.9694	− 58.4119	− 54.0375	− 64.6093	− 58.5245	− 59.7577
Ethyl 2-oxopropanoate	− 62.6881	− 73.4001	− 46.0156	− 55.4678	− 69.9755	− 64.1391	− 69.1988
(1,3-^13^C_2_)propanedioic acid	− 74.3819	− 88.8806	− 83.2791	− 74.408	− 106.82	− 76.2029	− 86.8522
2-butyl-4-[(E)-pentadec-4-enyl]-1,3,2-dioxaborinan-5-amine	− 49.5347	− 60.9228	− 47.6395	− 49.6682	− 56.4944	− 55.7986	− 48.7232
2,4-dimethyl-1,3-dioxane	− 75.7941	− 76.8513	− 77.4718	− 75.5358	− 87.5893	− 78.6818	− 81.3197
5-amino-6-nitroso-1*H*-pyrimidine-2,4-dione	− 58.8103	− 62.1977	− 59.1507	− 62.4201	− 74.5919	− 69.2034	− 64.595
(2*R*,4*S*,5*S*,6*R*)-3,8,9-trioxatricyclo[4.2.1.0^2,4^]nonan-5-ol	− 63.0445	− 64.2929	− 58.6933	− 64.2665	− 76.5104	− 77.3524	− 67.3094
(1*R*,2*S*,4*R*,5*R*)-3,7,9-trioxatricyclo[4.2.1.0^2,4^]nonan-5-ol	− 58.317	− 66.9296	− 57.4211	− 62.3883	− 74.6524	− 69.2782	− 64.748
(2*S*,4*S*,5*R*,6*R*)-3,8,9-trioxatricyclo[4.2.1.0^2,4^]nonan-5-ol	− 58.1171	− 58.2697	− 49.6619	− 53.7779	− 58.2695	− 59.8385	− 51.2265
5-methylhex-3-yn-2-ol	− 79.8391	− 85.5672	− 93.6498	− 62.3278	− 85.3802	− 88.8687	− 78.6849
Trimethylsilyl tetracosanoate	− 61.5327	− 59.2993	− 71.4039	− 60.1473	− 57.8295	− 79.5731	− 65.2117

In general, among the 25 phytocompounds, serverogenin acetate ([3,12-diacetyloxy-10,14-dimethyl-13-oxo-15-(5-oxo-2H-furan-3-yl)-2-oxapentacyclo[9.7.0.01,3.05,10.014,18]octadecan-7-yl] acetate) exhibited the best binding conformations with the lowest binding energy values with bacterial (− 103.14 kcal/mol), fungal (− 105.78 kcal/mol), viral (− 90.53 kcal/mol), antioxidant (− 82.85 kcal/mol) and diabetic [e.g. aldose reductase (− 137.03 kcal/mol), α-glucosidase (− 94.64 kcal/mol) and α-amylase (− 94.77 kcal/mol)] target proteins (Table 6). Our findings are supported by the results of a previous study that showed that the lower the binding energy score was found the better the protein–ligand binding stability was identified^24^.

**Figure 9:**
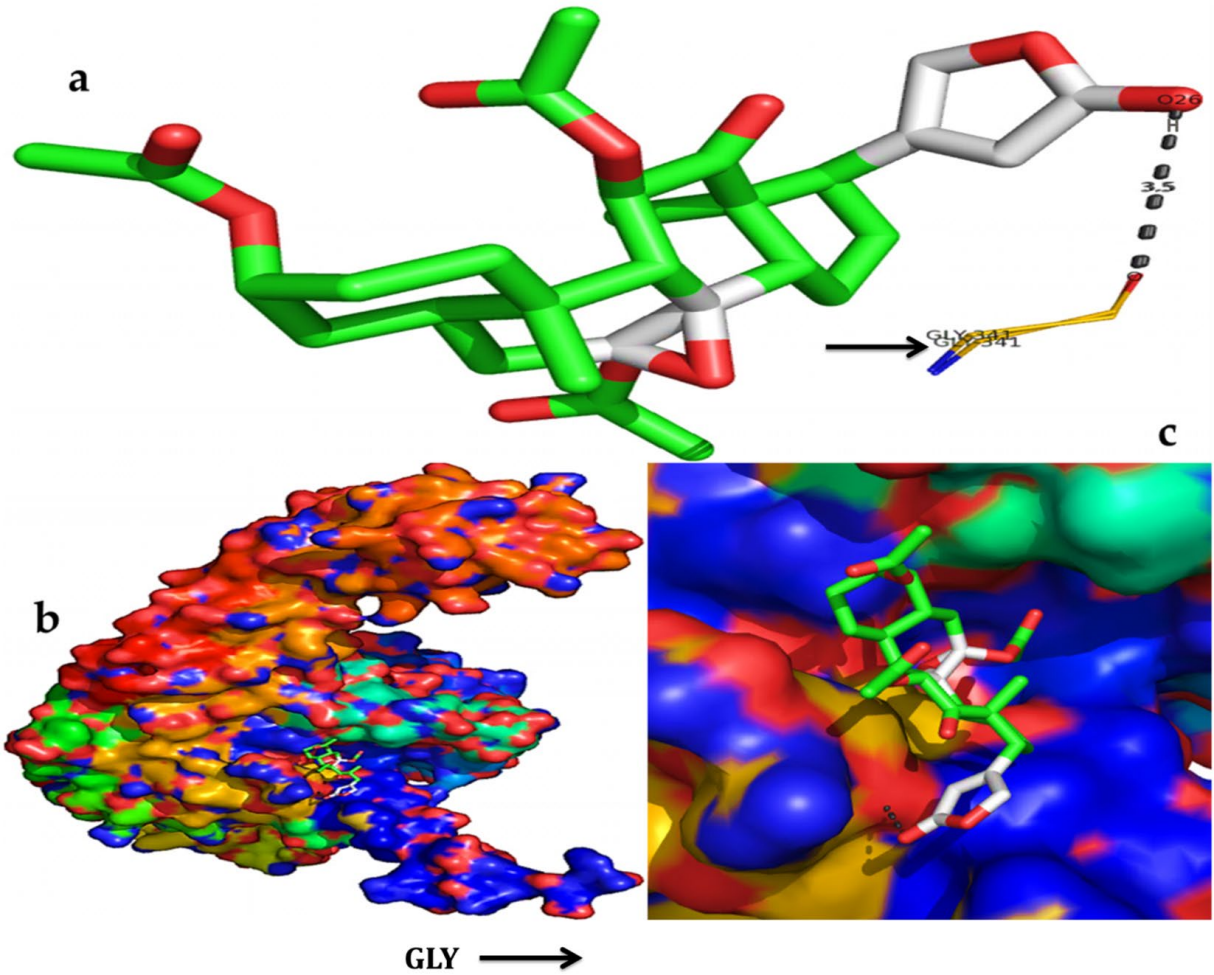
In silico molecular docking shows the binding interaction of serverogenin acetate ([3,12-diacetyloxy-10,14-dimethyl-13-oxo-15-(5-oxo-2H-furan-3-yl)-2-oxapentacyclo[9.7.0.01,3.05,10.014,18]octadecan-7-yl] acetate) compound with bacterial target protein (5iwm) based on the binding energy generated by AutoDock program. (**a**) A hydrogen-bond interaction is formed by O26 in GLY341 (black arrow) in the serverogenin acetate compound. (**b**) A close-up view of serverogenin acetate compound that binds to the bacterial target protein. (**c**) Binding pose of the bacterial target protein with serverogenin acetate compound.

**Figure 10:**
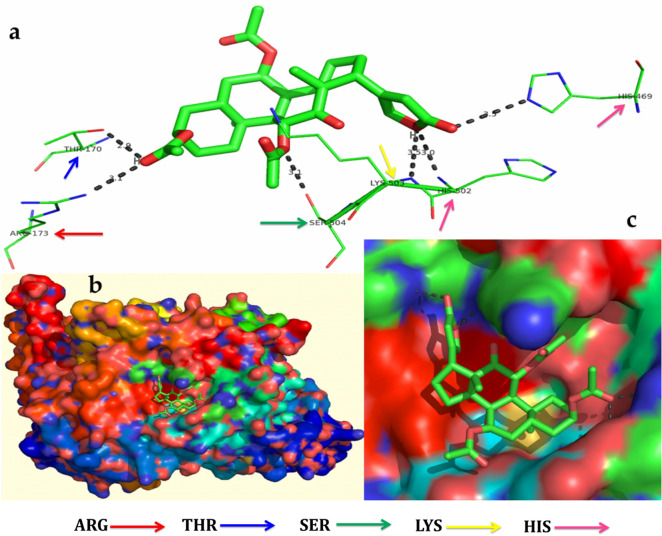
In silico molecular docking shows the binding interaction of serverogenin acetate ([3,12-diacetyloxy-10,14-dimethyl-13-oxo-15-(5-oxo-2H-furan-3-yl)-2-oxapentacyclo[9.7.0.01,3.05,10.014,18]octadecan-7-yl] acetate) compound with fungal target protein (4i9p) based on the binding energy generated by AutoDock program. (**a**) Hydrogen-bond interaction are formed by THR170 (blue arrow), ARG173 (red arrow), SER504 (green arrow), LYS503 (yellow arrow), HIS502 and HIS469 (pink arrow) in the serverogenin acetate compound. (**b**) A close-up view of serverogenin acetate compound that binds to the fungal target protein. (**c**) Binding pose of fungal target protein with serverogenin acetate compound.

**Figure 11:**
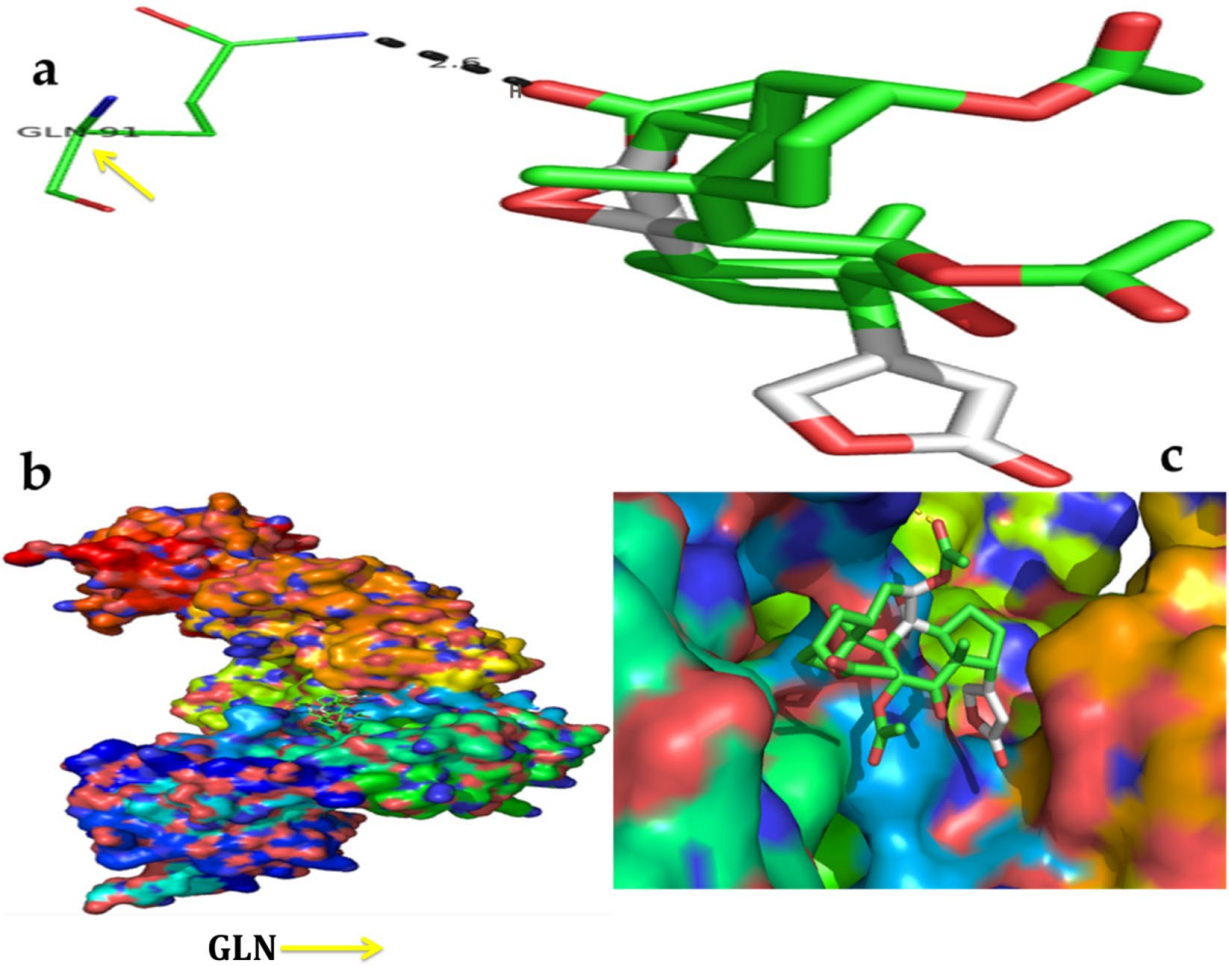
In silico molecular docking shows the binding interaction of serverogenin acetate ([3,12-diacetyloxy-10,14-dimethyl-13-oxo-15-(5-oxo-2H-furan-3-yl)-2-oxapentacyclo[9.7.0.01,3.05,10.014,18]octadecan-7-yl] acetate) compound with viral target protein (1rev) based on the binding energy generated by AutoDock program. (**a**) A hydrogen-bond interaction is formed by GLN91 (orange arrow) in the serverogenin acetate compound. (**b**) A close-up view of serverogenin acetate compound that binds to the viral target protein. (**c**) Binding pose of viral target protein with serverogenin acetate compound.

**Figure 12:**
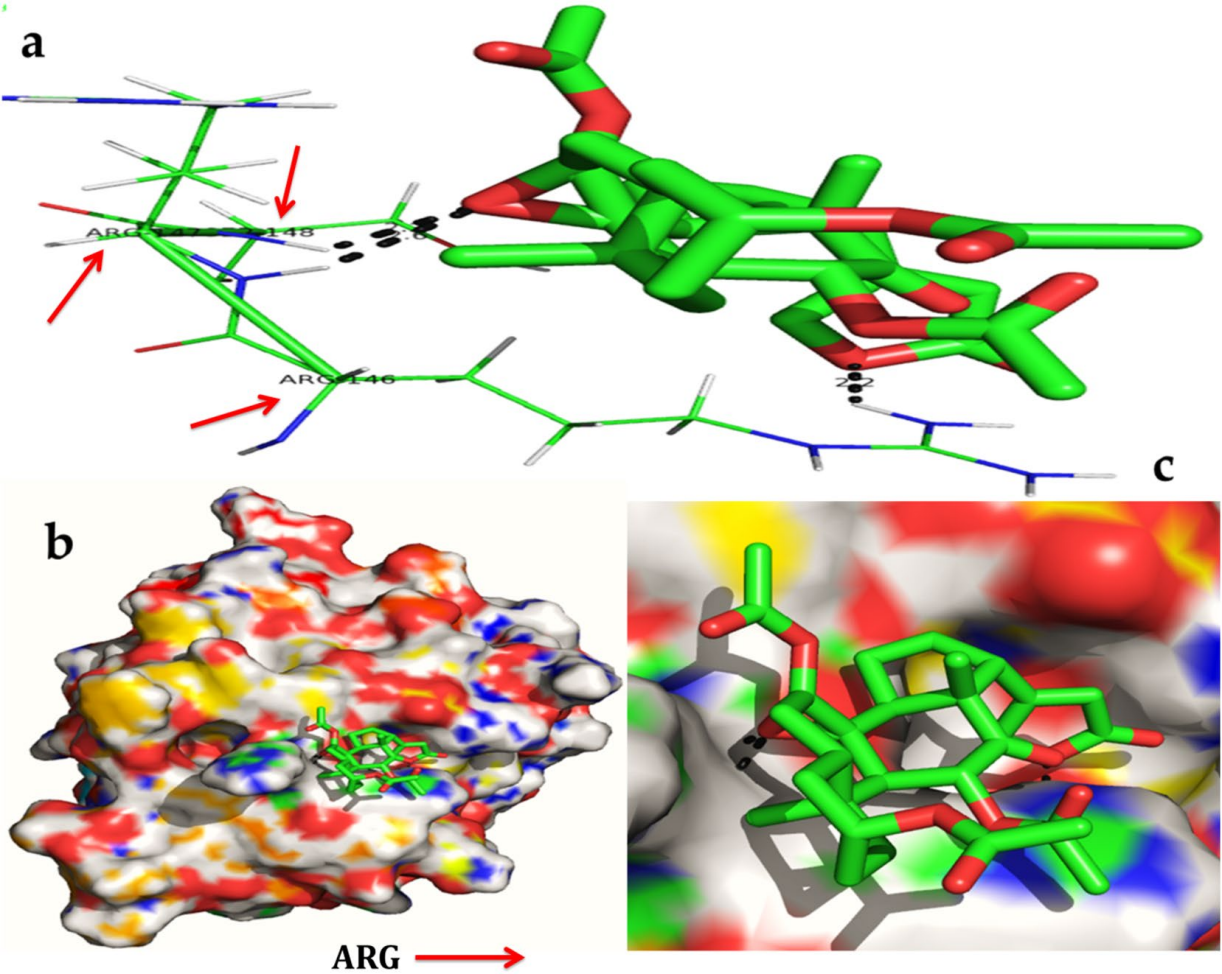
In silico molecular docking shows the binding interaction of serverogenin acetate ([3,12-diacetyloxy-10,14-dimethyl-13-oxo-15-(5-oxo-2H-furan-3-yl)-2-oxapentacyclo[9.7.0.01,3.05,10.014,18]octadecan-7-yl] acetate) compound with antioxidant target protein (2he3) based on the binding energy generated by AutoDock program. (**a**) A hydrogen-bond interaction is formed by O in ARG146, ARG147 and ARG148 (red arrow) in the serverogenin acetate compound. (**b**) A close-up view of serverogenin acetate compound that binds to the anti-oxidant target protein. (**c**) Binding pose of antioxidant target protein with serverogenin acetate compound.

**Figure 13:**
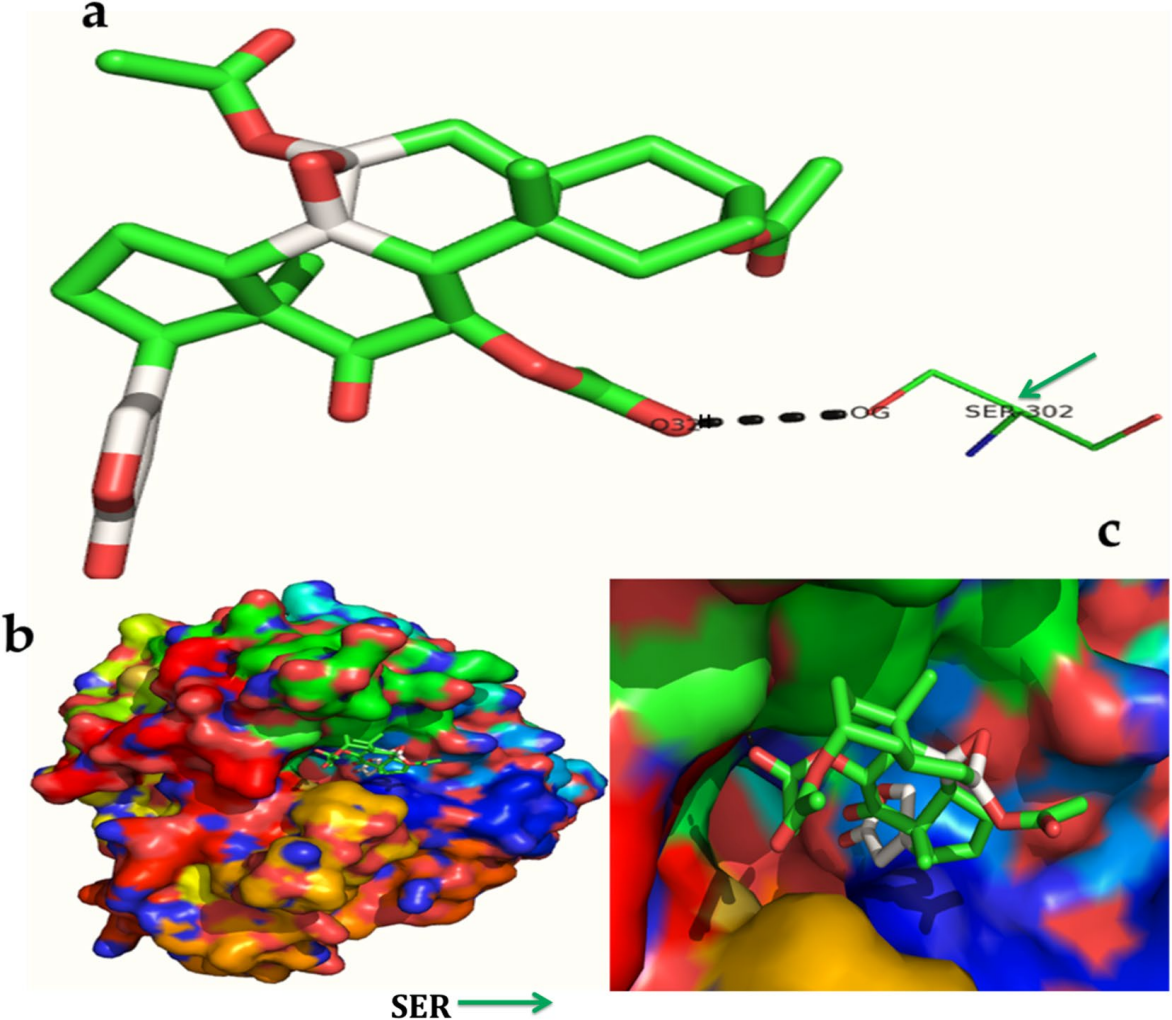
In silico molecular docking shows the binding interaction of serverogenin acetate ([3,12-diacetyloxy-10,14-dimethyl-13-oxo-15-(5-oxo-2H-furan-3-yl)-2-oxapentacyclo[9.7.0.01,3.05,10.014,18]octadecan-7-yl] acetate) compound with aldose reductase target protein (4ysl) based on the binding energy generated by AutoDock program. (**a**) A hydrogen-bond interaction is formed by O3 and OG in SER302 (green arrow) in the serverogenin acetate compound. (**b**) A close-up view of serverogenin acetate compound that binds to the aldose reductase target protein. (**c**) Binding pose of aldose reductase target protein with serverogenin acetate compound.

**Figure 14:**
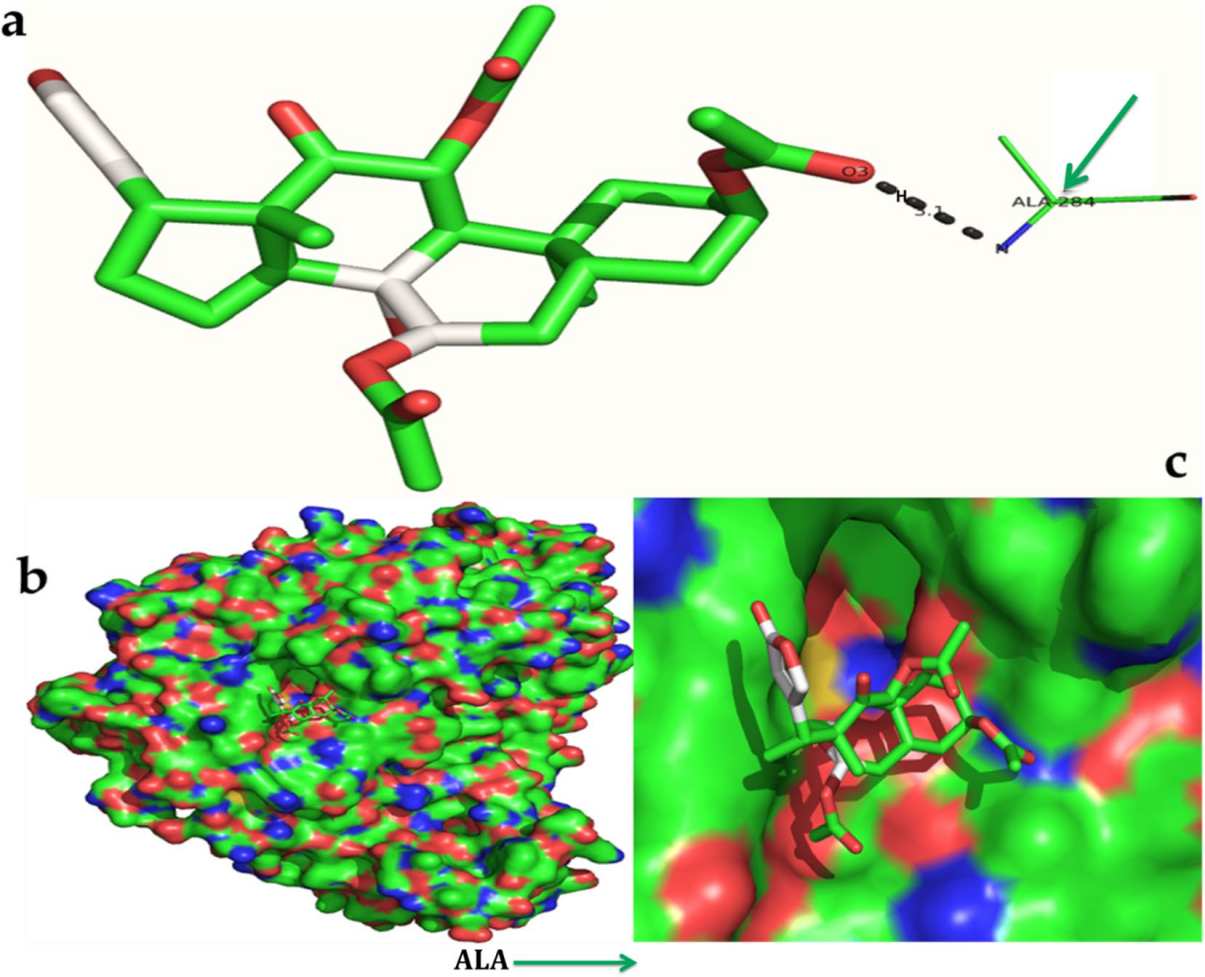
In silico molecular docking shows the binding interaction of serverogenin acetate ([3,12-diacetyloxy-10,14-dimethyl-13-oxo-15-(5-oxo-2H-furan-3-yl)-2-oxapentacyclo[9.7.0.01,3.05,10.014,18]octadecan-7-yl] acetate) compound with α-glucosidase target protein (5nn3) based on the binding energy generated by AutoDock program. (**a**) A hydrogen-bond interaction is formed by O3 in ALA284 (bright green arrow) in the serverogenin acetate compound. (**b**) A close-up view of serverogenin acetate compound that binds to the α-glucosidase target protein. (**c**) Binding pose of α-glucosidase target protein with serverogenin acetate compound.

**Figure 15:**
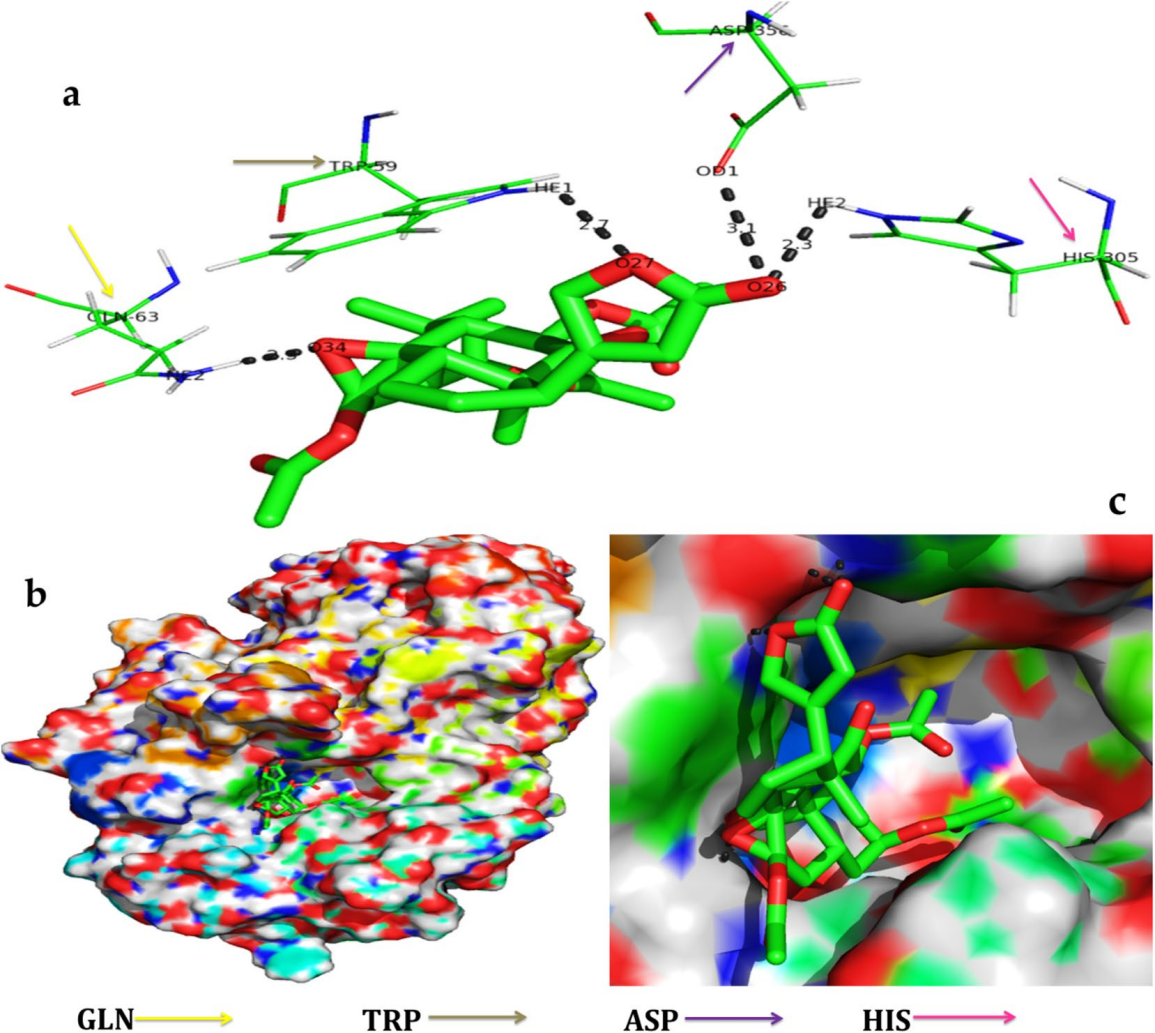
In silico molecular docking shows the binding interaction of serverogenin acetate ([3,12-diacetyloxy-10,14-dimethyl-13-oxo-15-(5-oxo-2H-furan-3-yl)-2-oxapentacyclo[9.7.0.01,3.05,10.014,18]octadecan-7-yl] acetate) compound with α-amylase target protein (i-tasser) based on the binding energy generated by AutoDock program. (**a**) A hydrogen-bond interaction is formed by O26 in HE2, O27 in HE1, O34 in HE2, TRP59 (grey arrow), GLN63 (orange arrow), HIS305 (pink arrow) and ASP356 (purple arrow) in the serverogenin acetate compound. (**b**) A close-up view of serverogenin acetate compound that binds to the α-amylase target protein. (**c**) Binding pose of α-amylase target protein with serverogenin acetate compound.

The serverogenin acetate formed the most excellent ligand–protein complexes (e.g. with six of seven tested target proteins) compared with other compounds. Next, docking analysis was then carried out for serverogenin acetate with the target proteins through AutoDock program. According to the binding energies, the docking results of serverogenin acetate with target proteins were ranked. Analysis of the docking results showed the potential strength of binding affinity of serverogenin acetate into the binding sites of target proteins with minimum binding energies (ranging from − 3.31 to − 4.22 kcal/mol), ligand efficiency (− 0.08 to − 0.11 kcal/mol), inhibition constant (1.01 to 804.76 µM) and Van der Waals (VDW) + hydrogen bonding (Hbond) + desolvation energy (− 4.17 to − 5.14 kcal/mol) (Table 7). This serverogenin compound also formed hydrogen-bond interactions and the best possible binding pose with the residues of bacterial (Fig. 9a-c), fungal (Fig. 10a-c), viral (Fig. 11a-c), antioxidant (Fig. 12a-c), aldose reductase (Fig. 13a-c), α-glucosidase (Fig. 14a-c) and α-amylase (Fig. 15a-c) target proteins, respectively as shown by their corresponding 3D interaction models.

**Table 7:** Automated docking binding interactions results of serverogenin acetate ([3,12-diacetyloxy-10,14-dimethyl-13-oxo-15-(5-oxo-2H-furan-3-yl)-2-oxapentacyclo[9.7.0.01,3.05,10.014,18]octadecan-7-yl] acetate) with target proteins.

Target protein	Binding energy (Kcal/mol)	Ligand efficiency (Kcal/mol)	Inhibition constant (µM)	Van der Waals + hydrogen bonding + desolvation energy (Kcal/mol)
Antibacterial 5iwm	− 4.22	− 0.11	804.76	− 4.9
Antifungal 4i9p	− 4.09	− 0.1	1.01	− 5.14
Antiviral 1rev	− 3.65	− 0.09	2.11	− 4.32
Antioxidant 2he3	− 3.31	− 0.08	3.75	− 4.17
Aldo reductase 4ys1	− 4.03	− 0.1	1.11	− 5.08
Alpha glucosidase 5nn3	− 4.03	− 0.1	1.11	− 4.79
Alpha amylase i-tasser	− 3.82	− 0.1	1.58	− 4.56

## Discussion

In the present study, the investigation of ethyl acetate and methanol extracts from leaves and methanol extract from rhizomes of *Amomum nilgiricum* revealed the presence of various phytoconstituents, including flavonoids, carbohydrates, anthocyanin, cardiac glycosides, tannins, phenols, amino acids, alkaloids, anthroquinone, steroids, proteins, terpenoids, leucoanthocyanin, phytosterols, saponins and diterpenes. These bioactive phytoconstituents could be responsible for the therapeutic ability of various extracts of *A. nilgiricum*. The analysis was carried out by gas chromatography-mass spectrometry (GC–MS), one of the most widely used techniques for separation of phytoconstituents. The GC–MS investigation of *A. nilgiricum* leaves and rhizome extracts revealed the presence of 25 phytochemical compounds, which could contribute to the medicinal properties of this plant species^16,25^. 5-(aminooxy)pentanoic acid has been reported to be present in ethanol stem and leaf extracts of *Saccharum munja* exhibiting antioxidant and antibacterial activities^26^. The phytocompound 3,7,9-trioxatricyclo[4.2.1.0^2,4^]nonane extracted from methanol seeds extract of red *Vitis vinifera* has been reported for its antimicrobial and anti-inflammatory activities^27^. The trimethyl-(2-trimethylsilylphenyl)silane is an aromatic hydrocarbon, and has been reported in aqueous extract of *Centella asiatica* displayed anticancer, antioxidant and antimicrobial activities^28^.

The cyclic compounds are unsaturated; and hence, play a key role in the antioxidant defense. 3,5-bis(trimethylsilyl)cyclohepta-2,4,6-trien-1-one is a ketone compound with antioxidant activity^29^, which has been isolated from methanol leaf extract of *Psidium guajava* and methanol leaf extract of *Syzygium alternifolim*^30^. The phytoconstituent 3,5-bis(trimethylsilyl)cyclohepta-2,4,6-trien-1-one which was identified from n-hexane seeds extract of *Garcinia kola*^31^ and ethanol leaf extract of *Aporosa lindleyana*^32^ so far there is no evidence of its biological activity^30^. Likewise, trimethyl-[[4-(trimethylsilylmethyl)phenyl]methyl]silane is an aromatic silica compound, reported from *Cassia auriculata* and *Cardiospermum halicacabum* ethanol leaf extracts^33^, however its biological activity has not yet documented^33^. On the other hand, phytocompound trimethyl-[4-[2-methyl-4-(4-trimethylsilyloxyphenyl)pent-4-en-2-yl]phenoxy]silane was previously isolated from ethanol leaf extract of *Wedelia trilobata*^34^, trimethyl-[4-[4-(4-trimethylsilyloxyphenyl)hexan-3-yl]phenoxy]silane a sterol compound isolated from solvent mixture of chloroform and water (6:4) leaf and stem extracts of *Paganum harmala*^35^ and hexane and acetone (8:2) seeds extract of *Butea monosperma*^36^ documented with broad spectrum of antimicrobial properties^34–36^. Similarly, 1,1,1,3,5,5,5-heptamethyltrisiloxane is a siloxane compound which has been isolated from methanol leaf extract of *C. italica*^37^, aqueous bark extracts of *Khaya grandifoliola* and *Enantia chlorantha*^38^ as well as from ethanol rhizome extract of *Dryopteris cochleata*^39^ exhibited anti-inflammatory and antimicrobial properties^38,39^. While, octadec-1-yne**,** an alkene compound has been reported from chloroform extract of *Spermadictyon suaveolens* flowers showed antibacterial activity^40^. On contrary, octadec-1-yne extracted from petroleum ether and ethyl acetate leaves extracts of *Leucaena leucocephala* from Malaysia^41^ did not possess any phytopharmaceutical properties. In an independent study, the monoterpeniod compound, 3,4-heptadien-2-one, 3-cyclopentyl-6-methyl- reported from hexane flower extract of *Acmella uliginosa*^42^ and from hydrophilic leaf and flower extracts of *Succisa pratensis*^43^ reported significant antibacterial and antifungal activities.

Serverogenin acetate is a bioactive compound known for its application in pharmacology, this bioactive compound isolated from methanol leaf extracts of *Trichilia connaroides* demonstrated broad spectrum of biological properties such as antioxidant, anti-insect, antimicrobial, anticancer and antiulcerogenic activities^44^. The trimethylsilyl derivative compound trimethyl-[4-(2,4,4-trimethylpentan-2-yl)phenoxy]silane identified from n-hexane seed extract of *G. kola*^31^, chloroform and ethanol leaf extracts of *Acacia karroo* and *Ziziphus mauritiana*^45^ produced significant antibacterial activity against clinical bacteria and also recorded anti-inflammatory, anticancer, analgesic and diuretic activities. Ethyl 2-oxopropanoate is an androstane natured compound reported from methanol leaves extract of *A. nilotica*^46^ and *Kamettia caryophyllata*^47^ showed potent bioactivities of acidifier, acidulant and inhibits production of uric acid^47^. (1,3-^13^C_2_)propanedioic acid has been isolated from methanol extract of *Alysicarpus monilifer* whole plant^48^ however its biological activity is yet to confirm. The flavonoid compound 2,4-dimethyl-1,3-dioxane known to possess anti-inflammatory, analgesic, antibacterial and antifungal activities was isolated from Arjunarishta, a modified ayurvedic medicine^49^. 5-amino-6-nitroso-1*H*-pyrimidine-2,4-dione from methanol leaf extract of *Stachytarpheta jamaicensis* capable of producing high multi therapeutic properties such as antioxidant, anti-arthritic, anti-inflammatory and bactericidal potentials^50^. (2*S*,4*S*,5*R*,6*R*)-3,8,9-trioxatricyclo[4.2.1.0^2,4^]nonan-5-ol is a sugar moiety natured compound identified from ethanol extract of aerial parts of *Crotalaria longipes*^51^ and ethanol extracts of whole plant of *Sarcostemma secamone*^52^ reported to be used as a preservative. In this study, the leaf and rhizome extracts of *A. nilgiricum* showed remarkable in vitro antibacterial and antifungal activities by suppressing their colony and growth rate. In support of this study, earlier our group reported the potential antimicrobial activities of rhizome extracts of *A. niligiricum*^53^. In addition we have also noticed remarkable antioxidant and antidiabetic activities from the leaf and rhizome extracts of *A. nilgiricum* which is the first kind of report from this study. Previously, the antibacterial and antioxidant activities using the essential oil extracted from *A. subulatum* was documented Shrestha^54^. Similarly, Sharma et al.^55^ reported the antioxidant activity by seed extract of *A. subulatum*.

From the above evidences, it can be elucidated that *A. nilgiricum* plant consists of enormous potential of pharmacological constituents, therapeutic phytocompounds responsible for various pharmacological actions like antimicrobial, antioxidant anti-inflammatory, antidiabetic, analgesic, antiaging, anticancer, hepatoprotective, hypercholesterolemic, antihistaminic, antiandrogenic, antifibrinolytic, diuretic, antiasthma activities, preservative etc. These major chemical compounds identified from different crude extracts are considered to be a part of plants’ defense systems and they may be grouped as protective compounds found in this plant referring to them as 'phytoanticipins' and 'phytoprotectants'^56^. Thus, the identification of a various phytochemical compounds from ethyl acetate and methanol extracts from leaves and rhizomes of *A. nilgiricum* display significant medicinal properties of the plant *A. nilgiricum*. Further studies like bio-prospecting are essential to support its biological properties and biological importance of these innovative bio-molecules will be interesting to be studied. To the best of our knowledge, this is the first report on GC and MS investigation of *A. nilgiricum* leaf and rhizome extracts.

Bioinformatics tools provide a great support to pharmaceutical companies in the process of drug discovery in a short duration of time with less cost using all primary information obtained from in vivo and in vitro analysis. In silico molecular docking in one of the greatest methods to determine new ligand for proteins of identified structure and thus play an important role in structure based drug discovery. Investigators worldwide use computer docking programs to discover and investigate the binding affinity for compounds that fit a binding site on the protein. The structure of the protein and ligand should be three dimensional^57^. The study and documentation of structural compounds from different medicinal plant species are gaining interest and importance. In the structure based drug design, molecular docking is generally used to predict and inter molecular complex between the drug compounds with its target protein^58^. In the present study, a total of 25 bioactive compounds were identified from *A. nilgiricum* leaf and rhizome extracts by GC–MS analysis and used for molecular docking studies. Further, the compounds were analyzed for bioactivity against the target proteins. Serverogenin acetate is the lead compound and exhibited antibacterial, antifungal, antiviral, antioxidant and antidiabetic activities. Computational simulation studies revealed that serverogenin acetate compound recorded better affinity with low binding energy in comparison with other compounds. The docking study results also showed that various energy sources are consistent and contribute to the overall strength to the binding interactions of serverogenin acetate for each target proteins.

## Conclusions

The present investigation was focused on identification of various bioactive compounds from the leaf and the rhizome extracts of *Amomum nilgiricum* for the first time by GC–MS analysis. These compounds are responsible for the different therapeutic and pharmacological properties. We have also provided the evidence of leaves and rhizome extracts of *A. nilgiricum* for its antimicrobial (phytopathogens), antioxidant and antidiabetic activities. The serverogenin acetate ([3,12-diacetyloxy-10,14-dimethyl-13-oxo-15-(5-oxo-2H-furan-3-yl)-2-oxapentacyclo[9.7.0.01,3.05,10.014,18]octadecan-7-yl] acetate) compound showed promising binding affinity toward different proteins in molecular docking experiments, and their drug-like features were demonstrated through the iGEMDOCK docking analysis. Using the serverogenin acetate compound may enable us to develop an effective drug against pathogenic bacteria, fungi and viruses, as well as diabetic diseases. Further investigations to determine its bioactivity, toxicity profile and clinical studies are necessary for broad-spectrum drug discovery.

## Methods

### Collection and preparation of plant materials

The fresh leaves and rhizomes of *Amomum nilgiricum* (V.P. Thomas & M. Sabu) were collected from Western Ghats region of Palakkad district Kerala, India (11° 03′ 15.46" N, 076° 32′ 23.58" E) at altitude of 1150 m above mean sea level during 2017. The freshly collected leaves were thoroughly washed in running tap water and rinsed in distilled water before they were cut into small bits, then shade-dried at room temperature for 10 days, powdered using laboratory blender and preserved in 1000 ml air tight bottle. The dry leaf powder samples (100 g/each) were individually extracted with ethyl acetate and methanol solvents (500 mL) in a Soxhlet apparatus for 8 h respectively at a temperature not beyond the boiling point of the solvents. The ethyl acetate and methanol extracts were filtered using Whatman No. 1 filter paper, and the filtrates were concentrated under reduced pressure with a rotary evaporator, freeze-dried in a lyophilizer, and then kept at 4 °C. Similarly, the freshly collected rhizomes were thoroughly washed in running tap water and rinsed in distilled water. Subsequently, the samples were cut into small pieces, shade-dried at room temperature for 10 days, powered and extracted with methanol in a Soxhlet unit for 8 h. The extracted samples were filtered by Whatman filter paper, and the filtrates were concentrated at reduced pressure with a rotary evaporator, dried using a lyophilizer and kept at 4 °C until further use.

### Preliminary phytochemical screening

The qualitative phytochemical prescreening of leaf and rhizome extracts was performed following the previously published protocols^59^. The standard solution was prepared from 100 mg leaf or rhizome extract by dissolving the extract in 10 mL ethyl acetate or methanol. These solutions were then screened for the presence of different phytochemicals; namely, phenols, carbohydrates, flavonoids, anthocyanin, cardiac glycosides, saponins, tannins, alkaloids, steroids, terpenoids, amino acids, leucoanthocyanin, anthroquinone, proteins, phytosterols and diterpenes.

### Fourier transform infrared spectrometer (FTIR)

The FT-IR was performed with resolution in the spectral area of 4000–400 cm^−1^ to detect the possible functional groups. 10 mg of the dried *A. nilgiricum* leaf and rhizome extracts powder was encapsulated in 100 mg of KBr salt pellet, using a mortar and pestle, compressed into a thin pellet^13^.

### Gas chromatography-mass spectrometry (GC–MS) analysis

GC–MS analyses of leaf and rhizome extracts were carried out using the Perkin-Elmer Clarus 680 system (Perkin-Elmer, Inc. U.S.A) equipped with a fused silica column, packed with Elite-5MS) capillary column (30 m in length × 250 μm in diameter × 0.25 μm in thickness). Pure helium gas (99.99%) was used as the carrier gas at a constant flow rate of 1 mL/min. For GC–MS spectral detection, an electron ionization energy method was adopted with high ionization energy of 70 eV (electron Volts) with 0.2 s of scan time and fragments ranging from 40 to 600 m/z. The injection quantity of 1 μL was used (split ratio 10:1), and the injector temperature was maintained at 250 °C (constant). The column oven temperature was set at 50 °C for 3 min, raised at 10 °C per min up to 280 °C, and final temperature was increased to 300 °C for 10 min. The contents of phytochemicals present in the test samples were identified based on comparison of their retention time (min), peak area, peak height and mass spectral patterns with those spectral database of authentic compounds stored in the National Institute of Standards and Technology (NIST) library^60^.

### Molecular docking

All the compounds identified by GC–MS analysis were analyzed to study their putative antibacterial, antifungal, antiviral, antioxidant and antidiabetic activities through docking studies. The structures of the compounds were obtained from Pubchem database (https://pubchem.ncbi.nlm.nih.gov/) as sdf file and converted into Mol, PDBQT and PDB file formats using OPEN BABEL software (https://openbabel.org/wiki/Main_Page). The three-dimensional (3D) structures of phytocompounds were optimized for docking conformation study. The structure-based molecular docking was performed to study the protein ligand interactions of the phytocompounds with bacterial (PDB code:5iwm), fungal (PDB code:4i9p), viral (PDB code:1rev), antioxidant (PDB code:2he3) and diabetic [α-glucosidase (PDB code:5nn3), α-amylase (PDB code:i-tasser) and aldose reductase (PDB code:4ys1)] target proteins obtained from the Research Collaboratory for Structural Bioinformatics (RCSB) Protein Data Bank (https://www.rscb.org)^61^ using the iGEMDOCK 2.1 software (https://gemdock.life.nctu.edu.tw/dock/igemdock.php)^62^ and AutoDock program^63^.

Initially, all the target proteins and compounds were prepared by assigning hydrogen bonds, bond orders, charges and flexible torsions. Screening of these compounds for protein ligand interactions was carried out using the iGEMDOCK program with the preset parameters: population size of 150 was set with 60 generations and two solutions were selected for customized docking parameter. From the customized docking study, the probable binding conformations of phytocompounds were determined based on the iGEMDOCK total energy.

The best binding conformation of phytocompounds against target proteins were determined based on the lowest total binding energy among the different conformations generated. The identified phytocompounds were imported into the iGEMDOCK graphical user interface and were sorted by the post-docking analysis based on their binding energies and compound fitness score measured by the iGEMDOCK docking algorithm^64^. To determine the relative strengths of the binding interactions of the best identified phytocompound, screening for its best binding pose and the series of energy values, such as binding energy, ligand efficiency, inhibition constant and Van der Waals (VDW) + hydrogen bonding (Hbond) + desolvation energy of each target protein, were analyzed using the AutoDOCK program. Furthermore, the detailed interactions between the best phytocompound identified, and its binding sites with bacterial, fungal, viral and diabetic target proteins were visualized in PyMol 3D visualization^65^.

### Antimicrobial activity

Antimicrobial activity of leaf and rhizome extracts was determined using the agar well diffusion method. The antibacterial activity of leaf and rhizome extracts of *A. nilgiricum* was carried out against *Ralstonia solanacearum* (RS5-KF924743), *Staphylococcus aureus* (NCIM 2079), *Pseudomonas aeruginosa (*NCIM 2200)*, Escherichia coli* (NCIM 2256), *Klebsiella pneumonia* (NCIM 2957)*, Bacillus subtilis* (NCIM 2724), *Salmonella typhimurium* (NCIM 2501), and *Proteus vulgaris* (NCIM 2027) which were procured (except *R. solanacearum*) from National Collection of Industrial Microorganism (NCIM), NCL, Pune, India. Streptomycin (30 µg/mL) was used as a positive control. The antifungal activity of leaf and rhizome extracts of *A. nilgiricum* was carried out against *Aspergillus flavus* (NCIM 1028), *Aspergillus terreus* (NCIM 1325), *Aspergillus niger* (NCIM 1196), *F. oxysporum* (NCIM 1281), *Alternaria alternata* (NCIM 718) and *Pyricularia oryzae* were obtained from the NCIM, NCL, Pune India. Nystatin was used as a positive control.

### Antioxidant activity

The 2,2-diphenyl-1-picryl-hydrazyl-hydrate (DPPH) is a generally used method to evaluate the free radical scavenging ability of natural compounds. The free radical scavenging activity of the leaf and rhizome extracts of *A. nilgiricum* were measured against DPPH^66^. The Ferric reducing antioxidant power assay (FRAP) was used to measure the total antioxidant power from the leaf and rhizome extracts^67^.

### Antidiabetic activities

The α-amylase inhibition assay from *A. nilgiricum* leaf and rhizome extracts was determined using the 3,5-dinitrosalicylic acid method^68^. The α-glucosidase inhibition activity of *A. nilgiricum* leaf and rhizome extracts was measured using the method of Eom et al.^69^. Acarbose was used as positive control. The aldose reductase (AR) inhibition assay from *A. nilgiricum* leaf and rhizome extracts was determined according to Suryanarayana et al.^70^ using Quercetin as positive control. The inhibitory action was stated as the half maximal inhibitory concentration (IC_50_), which is a measure of the effectiveness of the extract in inhibiting enzymes activities.
